# Complete Genome Sequence of ER2796, a DNA Methyltransferase-Deficient Strain of *Escherichia coli* K-12

**DOI:** 10.1371/journal.pone.0127446

**Published:** 2015-05-26

**Authors:** Brian P. Anton, Emmanuel F. Mongodin, Sonia Agrawal, Alexey Fomenkov, Devon R. Byrd, Richard J. Roberts, Elisabeth A. Raleigh

**Affiliations:** 1 New England Biolabs, Inc., Ipswich, Massachusetts, United States of America; 2 Institute for Genome Sciences, University of Maryland School of Medicine, Baltimore, Maryland, United States of America; Louisiana State University and A & M College, UNITED STATES

## Abstract

We report the complete sequence of ER2796, a laboratory strain of *Escherichia coli* K-12 that is completely defective in DNA methylation. Because of its lack of any native methylation, it is extremely useful as a host into which heterologous DNA methyltransferase genes can be cloned and the recognition sequences of their products deduced by Pacific Biosciences Single-Molecule Real Time (SMRT) sequencing. The genome was itself sequenced from a long-insert library using the SMRT platform, resulting in a single closed contig devoid of methylated bases. Comparison with K-12 MG1655, the first *E*. *coli* K-12 strain to be sequenced, shows an essentially co-linear relationship with no major rearrangements despite many generations of laboratory manipulation. The comparison revealed a total of 41 insertions and deletions, and 228 single base pair substitutions. In addition, the long-read approach facilitated the surprising discovery of four gene conversion events, three involving rRNA operons and one between two cryptic prophages. Such events thus contribute both to genomic homogenization and to bacteriophage diversification. As one of relatively few laboratory strains of *E*. *coli* to be sequenced, the genome also reveals the sequence changes underlying a number of classical mutant alleles including those affecting the various native DNA methylation systems.

## Introduction

**Fig 1:**
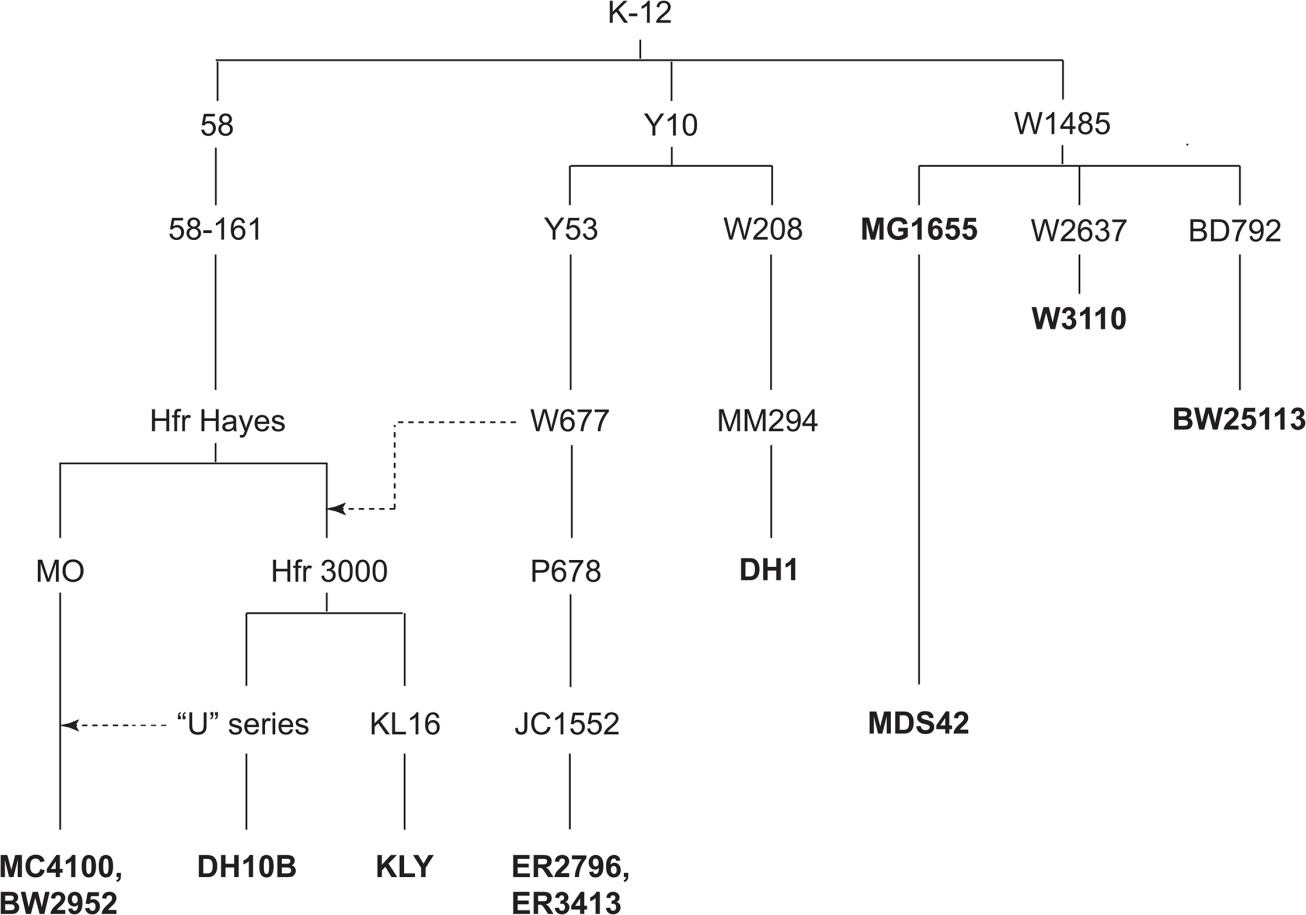
Relationship of ER2796 and ER3413 to the nine other completely sequenced *E*. *coli* K-12 strains. Completely sequenced strains are shown in bold type, and selected ancestral strains in Roman type. Most of the tree has been abstracted from Bachmann [28], except for the ancestries of MC4100 and DH10B back to Hfr Hayes, which are based on Laehnemann [63] and Durfee [4], respectively. Selected additional contributions of genetic material via crosses are shown by dotted lines. It appears based on genotype that Hfr 3000 U482 is the “U series” ancestor of DH10B, while Hfr 3000 U169 contributed genetic material in the ancestry of MC4100.

The Gram-negative bacterium *Escherichia coli* has been foundational to our understanding of bacterial genetics since 1946, when Lederberg and Tatum first demonstrated bacterial conjugation [1]. Between that time and the start of the genome sequence era one half century later, a wealth of *E*. *coli* genotypic and phenotypic information was generated through laboratory manipulation of strains. Currently, there are finished genome sequences available for more than 75 *E*. *coli* strains, but the vast majority of these are wild type isolates, both pathogenic and commensal. The few laboratory strains that have been completely sequenced include nine strains derived from K-12 (Table 1 and Fig 1). Two of these strains (MG1655 and W3110) resulted from only minimal genetic manipulation of the wild type K-12 isolate [2, 3] and are highly similar to one another [3]. The other seven strains have been more extensively manipulated, and provide some insight into the nature of both classical mutant alleles and previously unidentified lineage-specific mutations [4, 5].

**Table 1:** Laboratory strains of *E*. *coli* with finished (ungapped) genome sequences in GenBank.

Strain	Ancestor	RefSeq or *INSDC* Accession (chromosome)	Reference
MG1655	K-12	NC_000913	[2, 64]
W3110	K-12	NC_007779	[3]
DH1	K-12	NC_017625, NC_017638	[65]
DH10B	K-12	NC_010473	[4]
BW2952 [MC4100(MuLac)]	K-12	NC_012759	[5]
MDS42	K-12	NC_020518	unpublished
MC4100	K-12	*HG738867*	[63]
BW25113	K-12	*CP009273*	unpublished
KLY	K-12	*CP008801*	[66]
ER2796	K-12	*CP009644*	this work
ER3413	K-12	*CP009789*	this work
REL606	B	NC_012967	[67]
BL21(DE3)	B	NC_012971, NC_012892	[67]
BL21-Gold	B	NC_012947	unpublished
W (ATCC 9637)	W	NC_017635, NC_017664	[68, 69]
KO11	W	NC_017660, NC_016902	[69]
LY180	W	NC_022364	unpublished
ATCC 8739	Crooks	NC_010468	unpublished

Some important genetic markers not present in any of the sequenced strains above relate to DNA methylation. *E*. *coli* K-12 encodes four DNA methyltransferases (MTases), one of which forms part of a Type I restriction-modification (R-M) system and three of which are solitary (Table 2). Methylation of GATC sites by M.EcoKDam has been well studied and is known to serve several important functions including directing mismatch repair to the nascent strand, regulating the timing of chromosome replication, and controlling the expression of certain genes (reviewed in [6]). Methylation of CCWGG by M.EcoKDcm is less well understood, but the *dcm* gene partially overlaps *vsr*, which encodes the very short patch (VSP) repair endonuclease (ENase), suggesting the two gene products function in a common process. Indeed, the VSP repair system fixes T:G mismatches that arise from the deamination of 5-methylcytosine (m^5^C), the product of Dcm methylation [7]. The biological function of Dcm methylation remains unclear, but recent evidence suggests it plays a role in controlling the expression of certain genes in stationary phase [8]. In particular, Dcm methylation appears to downregulate the expression of ribosomal genes [9], possibly by regulating *rpoS* expression [8]. The MTase M.EcoKII, encoded by the gene *yhdJ*, has been shown to methylate the site ATGCAT at the second A residue when overexpressed from a plasmid copy, conferring protection from the restriction enzyme NsiI [10]. However, this activity has yet to be observed in wild type cells, suggesting the gene is silent under all growth conditions tested to date. Its biological function remains unknown despite its wide conservation in almost all sequenced *E*. *coli* genomes. The MTase encoded by *hsdM* forms part of the Type I R-M system EcoKI, and methylates both strands of the asymmetric sequence AACNNNNNNGTGC. In Type I R-M systems, specific DNA recognition by the MTase (M) is not intrinsic, but rather is conferred by a specificity subunit (S), with the active methylation complex having the stoichiometry M_2_S [11].

**Table 2:** DNA restriction-modification genes in *E*. *coli* K-12 MG1655.^a^

	Gene	Product	Activity
DNA MTases			
	*dam*	orphan MTase M.EcoKDam	G^m6^ATC
	*dcm*	orphan MTase M.EcoKDcm	C^m5^CWGG
	*yhdJ*	orphan MTase M.EcoKII	ATGC^m6^AT (silent)
	*hsdM*	Type I MTase M.EcoKI	A^m6^ACNNNNNNGTGC
Other Genes			
	*hsdR*	Type I restriction ENase R.EcoKI	
	*hsdS*	Type I specificity subunit S.EcoKI	
	*mcrA*	Type IV restriction ENase	
	*mcrBC*	Type IV restriction ENase	
	*mrr*	Type IV restriction ENase	

^a^ All of these genes have been deleted or otherwise inactivated in ER2796 except for *yhdJ*, which is additionally inactivated in ER3413.

None of the four MTases are essential for viability. R-M systems are exchanged primarily by horizontal gene transfer, and the EcoKI system lies in a highly plastic region of the genome referred to as the immigration control region (ICR) [12]. The general variability of this region between *E*. *coli* strains [13] suggests this region can be removed without significant consequence, and consistent with this, the sequenced strain DH10B shows the Δ(*mrr-hsdRMS-mcrBC*) allele to be a deletion of a block of 45 genes including the entire ICR as well as flanking regions [4]. The gene *yhdJ* has also been deleted with no detectable phenotype, which is not surprising given that it appears to be silent under normal conditions [10]. Inactivating mutations of *dcm* also have no visible phenotype, although VSP repair is lost in at least one allele [14] and there are clearly alterations in the expression patterns of certain genes [8, 9]. *E*. *coli dam* mutant strains are viable, but show a pleiotropic effect with most phenotypes attributable to loss of strand discrimination during mismatch repair. These include increased rates of mutation [15] and recombination [16], increased sensitivity to certain cytotoxic agents such as cisplatin [17] and alkylating agents [18], widespread alteration of gene expression patterns [19] including activation of genes in the SOS regulon [20], and an absolute requirement for recombination (*recA*, *recB*, *recC*, *recG*, *ruvA*, *ruvB*, and *ruvC* genes) due to accumulation of double-strand DNA breaks [20–22].

Strains deficient in one or more of these MTases, particularly the *dam* and *dcm* functions, have found important uses in the molecular biology laboratory [14]. Since *E*. *coli* is the primary host for propagating plasmid DNA in the laboratory, and most *E*. *coli* strains are Dam^+^/Dcm^+^, plasmids typically bear methylation at GATC and CCWGG sites. This methylation can interfere with digestion by REases whose recognition sites overlap with or contain these patterns, and so propagation in a Dam^–^/Dcm^–^ strain prior to digestion by such enzymes is required in these cases. In addition, methylation can trigger cleavage of DNA by Type IV (methyl-dependent) R-M systems, greatly reducing transformation efficiency of methylated plasmids in bacteria containing such systems [23]. Propagation in a Dam^–^/Dcm^–^ strain to erase methylation patterns prior to transformation can alleviate this problem [24]. Older methodologies such as site-directed mutagenesis and Maxam-Gilbert DNA sequencing also benefited from Dam^–^/Dcm^–^ strains [14], but these have largely been supplanted by newer technologies.

One emerging technology that benefits from methyl-deficient *E*. *coli* strains is Single-Molecule Real-Time (SMRT) DNA sequencing. Recent studies have shown that this sequencing technology can easily detect DNA methylation sites on both plasmid clones [25] and whole chromosomes [26], and has thus enabled the facile determination of recognition sites of DNA MTases. These patterns are most obvious when not obscured by *dam* and *dcm* methylation, and so the *E*. *coli* strain used for cloning DNA MTase genes in both of these studies was ER2796 (also called DB24 [27]), in which all three active, endogenous MTases have been inactivated (*dam dcm hsdM*), to ensure that the cloned heterologous MTase is solely responsible for any methylation observed. In this work we describe the complete genome sequence of ER2796.

## Materials and Methods

### Construction of ER2796

**Fig 2:**
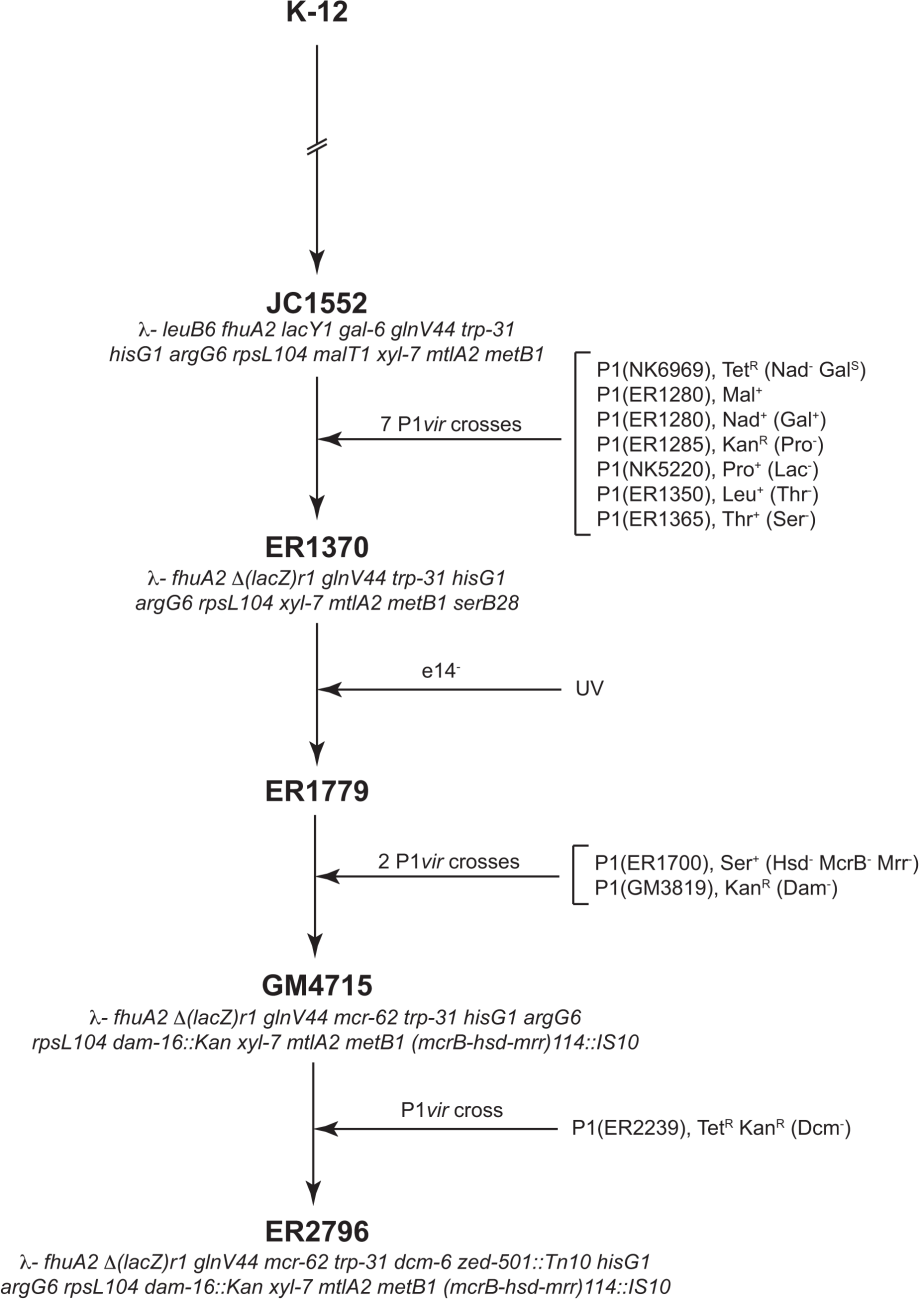
Lineage showing the construction of ER2796 from JC1552. Selected intermediate genotypes are shown. Markers that were selected are shown, followed by those that were screened for in parentheses. The lineage from K-12 to JC1552 has been described previously [28]. Genotypes are shown here using the historic allele names, but we suggest an updated nomenclature for some of these in Table 3 based on the genome sequence.

ER2796 is derived from JC1552, whose lineage from K-12 has been described previously (reference [28], with a condensed version shown in Fig 1). The construction of JC1552 involved, at various stages, treatment with X-ray and UV radiation, nitrogen mustard, and ethyl methanesulfonate, as well as a single conjugative cross with another K-12 derivative. Fig 2 shows the derivation of ER2796 from JC1552. In brief, strain ER1370 was derived from JC1552 by a series of P1*vir* crosses; strain ER1779 was derived from ER1370 by UV treatment, which inactivated *mcrA* restriction, retrospectively attributed to loss of the e*14* prophage; and ER2796 was derived from ER1779 again by P1*vir* crosses, which deleted the restriction cluster (including *hsdM*) and introduced inactivating alleles of *dam*, and *dcm*. The intermediate strain GM4715, and the strain GM3819 used for the *dcm*-inactivating P1*vir* cross, have been described previously [14].

### Genomic DNA and library preparation

100 mL of an overnight culture of ER2796, grown in LB medium [29], was resuspended in 10 mL [50 mM Tris (pH 8.0), 1 mM EDTA, 25% sucrose]. To this was added 8 mL 10 mg/mL chicken egg white lysozyme (Sigma-Aldrich, St. Louis, MO) dissolved in [250 mM Tris (pH 8.0), 250 mM EDTA]. Cells were incubated with the lysozyme at 37°C for 2 hrs, followed by two freeze-thaw cycles in dry ice/ethanol to facilitate cell breakage. To this was added 12 mL [50 mM Tris (pH 8.0), 62.5 mM EDTA, 1% Triton X-100] to complete breakage. Lysed cells were extracted once with 30 mL Tris-buffered phenol and once with 30 mL methylene chloride. Roughly 17 mL of the top layer was recovered. DNA was precipitated by addition of 0.1 volumes of 5 M NaCl and 0.7 volumes isopropanol, washed twice with 70% ethanol, and resuspended in a total of 0.8 mL buffer TE (Qiagen, Germantown, MD). Recovery was 93 μg as measured on a Qubit fluorometer (Invitrogen, Carlsbad, CA). All mixing was performed by gentle inversion to minimize DNA breakage.

To remove RNA, 15 μg ER2796 genomic DNA was incubated with 100 units of RNase If (New England Biolabs, Ipswich, MA) at 37°C for 1 hr in a 150 μL volume in the manufacturer’s recommended buffer. DNA was sheared using a g-TUBE (Covarys, Woburn, MA) following the manufacturer’s recommendations for 20 kb fragments (one 60 s pass at 5800 rpm in an Eppendorf 5415 microcentrifuge). DNA was purified using the PowerClean DNA Cleanup Kit (MoBio, Carlsbad, CA) and resuspended in a total of 75 μL manufacturer’s buffer 7. Recovery was 5.7 μg as measured on a Qubit fluorometer. Analysis on a Bioanalyzer 2100 (Agilent Technologies, Lexington, MA) using a DNA-12000 chip showed a median fragment size of 10.8 kb.

A sequencing library was prepared using the DNA Template Prep Kit 2.0 (3–10 kb) (Pacific Biosciences, Menlo Park, CA) according to the manufacturer’s protocol for 20 kb template preparation with BluePippin size-selection. Input was 66 μL (5 μg) sheared DNA, and the final elution step was in 31 μL of the manufacturer’s Elution Buffer. Recovery was 1.7 μg DNA as measured on a Nanodrop ND-1000 spectrophotometer (Thermo Fisher Scientific, Wilmington, DE). The entire library (30 μL) was size-selected using BluePippin (Sage Science, Beverly, MA) with a 4000 bp start. Eluate (40 μL) was collected and the well was washed with 0.1% Tween20 buffer (Sage Science), which was combined with the eluate. Size-selected DNA (total 100 μL) was purified by one 1x AMpure PB magnetic bead step (Pacific Biosciences) and eluted in 31 μL Elution Buffer (Pacific Biosciences). Recovery of the library was 730 ng at 23.7 ng/μL, as measured by Nanodrop spectrometry.

### DNA sequencing and assembly

Genome sequencing was carried out on the PacBioRS2 (Pacific Biosciences) using the DNA/Polymerase Binding Kit P4, MagBead Loading Kit, and Sequencing Kit 2.0 (all Pacific Biosciences). Data from 4 SMRT cells was used, with one 180 min movie per cell. Sequencing reads were assembled using the HGAP 2.0 program in Pacific Biosciences’ SMRTAnalysis pipeline. A mean coverage of 325x was achieved.

The sequence resolved into a single contig of 4,572,343 bp, of which roughly 13,700 bp was duplicated on the ends. Errors in the ends were analyzed by reassembling all reads against this duplicated region using the RS_Resequencing.1 program, and it was confirmed that all errors were confined to the 5’ end of the overlap at the start of the contig, and the 3’ end at the end of the contig, where coverage is lowest. The final non-redundant sequence was extracted to avoid the error-containing regions and rotated to bring the start in line with that of *E*. *coli* MG1655 (GenBank NC_000913.2).

### Genome annotation

Genome annotation of the 4,558,663 bp chromosome of ER2796 was performed using the Institute for Genome Sciences (IGS) Prokaryotic Annotation Pipeline (http://ae.igs.umaryland.edu/cgi/intro_info.cgi). Briefly, an initial set of open reading frames (ORFs) likely to encode proteins was identified by GLIMMER (http://cbcb.umd.edu/software/glimmer/), overlapping ORFs were removed, and the resulting set of ORFs was searched against a database of non-redundant protein sequences, nr (composed of non-redundant GenBank CDS translations + PDB + SwissProt + PIR + PRF, excluding those in env_nr), downloaded locally on the IGS systems. Two sets of hidden Markov models (HMMs) were used to determine ORF membership in families and superfamilies. These included 14,831 HMMs from PFAM version 27.0 (http://pfam.sanger.ac.uk/) and 4,284 HMMs from TIGRFam version 13.0 (http://www.jcvi.org/cgi-bin/tigrfams/index.cgi). TOPPRED was used to identify membrane-spanning domains in proteins.

### Identification of candidate gene conversion events

The list of single nucleotide polymorphisms (SNPs) was inspected for clusters of changes from MG1655 (>2/100 nt). Four such clusters were identified, all within repeated sequences. These segments, with flanking sequences, were used as probes to BLAST the NCBI Genomes TaxID 83333 (*E*. *coli* K-12 sequences NC_000913.3 [MG1655], NC_020518.1 [MDS42], NC_007779.1 [W3110], NZ_CM000960.1 [MG1655star], NC_012759.1 [BW2952], and NC_010473.1 [DH10B]), looking for 100% match. For each such cluster, at least one matching sequence was identified at a distant locus, as discussed below.

### Growth curves

Flasks of 25 mL Rich medium [29] with 100 μg/mL ampicillin were inoculated 1:250 with overnight cultures of MG1655 or ER2796 and grown at 37°C shaking at 225 rpm. At various time points, 200 μL of culture was withdrawn, diluted to 2 mL with Rich medium, and the OD_600_ of the diluted culture measured with a Biowave Cell Density Meter CO8000 (Biochrom Ltd., Cambridge, UK). Three replicate flasks were grown for each strain, and the mean of the readings was plotted. Growth rate constants were determined for the logarithmic growth phases (*t* = 50–159 min for MG1655, and *t* = 81–228 min for ER2796).

### Inactivation of YhdJ (M.EcoKII)

The wild type *yhdJ* (encoding M.EcoKII) was amplified by PCR from *E*. *coli* ER2796 using the forward primer TAGTTGCGAGCTCTTAAGGTTAACATATGAGAACAGGATGTGAACCGAC and reverse primer TTATTAGCATGCTTACTTTGTAATGAGATCGGGGTC. The resulting 885 bp product was digested with SacI and SphI (sites underlined) and subcloned into the respective sites of pUC19. The *yhdJ* ORF in the resulting clone was inactivated by digesting it at an internal AgeI site, filling in the 4-base overhangs using the Klenow fragment of DNA polymerase I, and religating the resulting blunt ends. We confirmed its inactivation by transforming *E*. *coli* ER2796 with the wild type and disrupted plasmids and digesting the genomic DNA with NsiI. As expected [10], the wild type clone conferred protection from NsiI digestion, while the inactivated clone did not.

We inactivated the chromosomal copy of *yhdJ* in ER2796 by allelic exchange using the suicide vector pRE112 (ATCC 87692) [30]. This plasmid contains a conditional R6K origin of replication, positive selectable marker encoding chloramphenicol resistance, and a derivative of the *Bacillus subtilis sacB* gene for counterselection on sucrose-containing media. We first subcloned the inactivated allele of *yhdJ*, described above, into pRE112 at the SacI and SphI sites and transformed the mobilizing donor strain *E*. *coli* S17-1 (a gift of the late Saul Roseman) with the resulting plasmid, called pRE112:M.EcoKIIΔAgeI. Transconjugation was performed by mixing S17-1 [pRE112:M.EcoKIIΔAgeI] with the original 2796 strain on an LB plate for 24 hours at 37°C. The accepting ER2796 strain is resistant to kanamycin (Km) and tetracycline (Tc), and the donor S17-1[pRE112:M.EcoKIIΔAgeI] strain only carries chloramphenicol (Cm) resistance from the pRE112-derived plasmid. Therefore, recombinant clones were selected on LB with Km, Tc and Cm. The pRE112:M.EcoKIIΔAgeI plasmid cannot replicate in ER2796, so the plasmid must integrate into the chromosome to confer Cm resistance. Site-specific recombinants were identified by colony PCR with *yhdJ* flanking primers TTAGTTGCTCTAGATTAAGGTTAACATATGTTCGAACAACGCGTAAATTCTGAC and TTATTAGCATGCATGGCAAAAAGAACCAAAGCCG.

Among 20 screened clones, only two (#9 and #18) demonstrated the correct site-specific insertion into the *yhdJ* locus (S1B Fig).

Through a second round of recombination, we selected for clones that had lost the vector backbone and replaced the wild type *yhdJ* allele with the inactivated one by growth on LB medium containing Km, Tc, and 5% sucrose. Cm-sensitive and sucrose tolerant clones were screened by PCR amplification with the *yhdJ* flanking primers and digestion with AgeI. All 10 of the screened clones were resistant to AgeI digestion (S1C Fig). Sequencing of the *yhdJ* region of one such clone confirmed the disruption of the ORF as the expected 4 bp insertion, and this strain was designated ER3413.

### Nucleotide accession numbers

The genome sequences of ER2796 and ER3413 are available from GenBank with the accession numbers CP009644 and CP009789, respectively. Both strains are available from New England Biolabs and the Coli Genetic Stock Center (CGSC).

## Results

### Inactivation of MTases

The three active, native *E*. *coli* MTases were all inactivated relatively recently in the lineage of ER2796, and all by P1*vir* crosses to introduce the mutant alleles (Fig 2). The *hsdM* gene, part of the EcoKI Type I RM system, is found in a cluster of restriction enzyme genes called the immigration control region (ICR). A deletion mutation (*Δ(fimB-opgB)114*::*IS10*) removing all of the restriction activities of this cluster was isolated following selection for loss of the ICR as in reference [31]. The deletion resulted from the action of the IS10 elements comprising the Tn10 insertion in *opgB* (formerly designated *zjj202*::*Tn10*). The IS10 action yielded an inversion/deletion event that removed adjacent DNA and left a tandem IS10 repeat at the position of the original insertion [32].

The inactivation of *dam* has been described previously [33]. Briefly, a 0.5 kb region of a plasmid copy of the gene was removed by digestion at unique EcoRV and HpaI sites, and a 1.1 kb fragment containing the Kan^R^ marker from Tn903 was inserted in its place. This defective copy, which conferred no detectable Dam methylation activity *in vitro* or *in vivo*, was then recombined onto the chromosome. Only the first 54 residues of the protein remain encoded by ER2796.

The inactivation of *dcm* has also been described [34]. Mutagenesis was accomplished by treatment of cultures with *N*-methyl-*N*-nitro-*N*-nitrosoguanidine, and mutants were screened for the ability to accept radiolabeled methyl groups using wild type crude extracts. It had been shown previously that the *dcm-6* null allele (which also results in loss of *vsr* activity) resulted from a nonsense mutation at the 45th codon [35], and our sequencing results confirm this in strain ER2796.

The disruption or loss of *dam*, *dcm*, and *hsdM* in ER2796 together render the strain free of DNA methylation under all known conditions. The remaining DNA MTase, *yhdJ*, is functional when cloned and overexpressed [10], but is silent in its native context for unknown reasons. Unlike most DNA MTase genes, which are horizontally acquired and quickly lost, *yhdJ* is well conserved among enteric bacteria, exhibiting a similar host range to *dcm*. Thus it seems likely that it has a biological role to play, and may be activated under specific conditions not yet identified. To guard against this possibility, we constructed the derivative strain ER3413 in which *yhdJ* was permanently inactivated by a 4 bp insertion in the coding sequence. Either of these two strains should provide a methylation-free background in which to observe the activities of exogenously introduced DNA MTases by SMRT sequencing.

### Sequencing and annotation of ER2796

**Fig 3:**
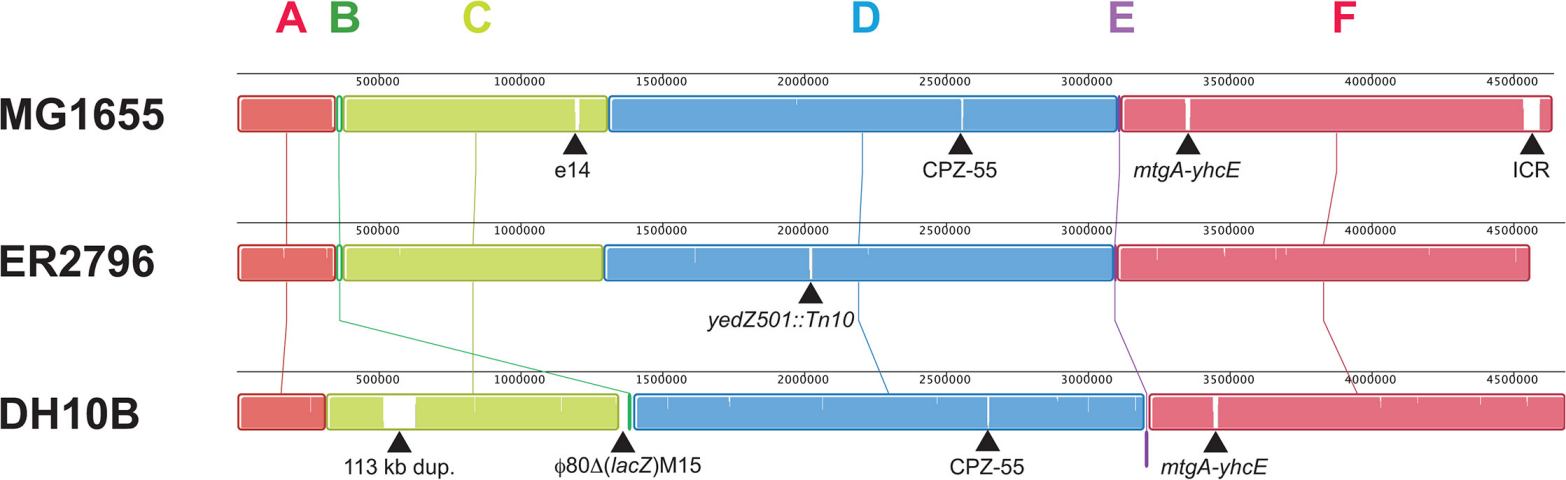
Alignment of the MG1655 genome with ER2796 and DH10B, conducted with Progressive-Mauve. Boundaries of the major contiguous blocks of sequence, labeled with capital letters, are formed by two major events specific to the DH10B lineage: block B results from deletion of a 34.6 kb region of MG1655 followed by partial restoration as part of a φ80Δ*(lacZ)M15* mosaic prophage insertion in DH10B; and block E results from the IS10-mediated inversion of an 11 kb segment of MG1655, again in DH10B [4]. The following larger indels visible in the figure are labeled: prophage e14 lost in both ER2796 and DH10B; prophage CPZ-55 lost in ER2796; the 16 kb *mtgA-yhcE* region lost in ER2796 through IS5-mediated deletion; the ICR region deleted in both ER2796 and DH10B; Tn10 insertion at *yedZ* in ER2796; tandem duplication of a 113 kb region in DH10B, presumably IS5-mediated; the φ80Δ*(lacZ)M15* mosaic prophage insertion in DH10B, including the *lacZ* region (part of block B).

We sequenced ER2796 using the Pacific Biosciences SMRT DNA sequencing platform from a large-insert library (see Materials and Methods), which resulted in a single linear contig. The closed circular genome is 4,558,663 base pairs in length and contains 50.8% G+C. Overall, the ER2796 genome shows high co-linearity with that of MG1655 (Fig 3). While ER2796 has undergone several insertions and deletions, there are no major rearrangements or inversions relative to MG1655.

Using the MG1655 annotation (NC_000913.2) as a template, we annotated a total of 4,083 protein-coding genes in ER2796. Genes annotated as “pseudogenes” in MG1655 were not re-annotated in ER2796. However, 31 genes intact in MG1655 are disrupted in some way (by frameshift, insertion, deletion, or nonsense mutation) in ER2796 and were annotated as new “pseudogenes” and included in the 4,083 total. (Not counted among these 31 are *hisG*, which suffered a small in-frame deletion, and *rph*, which suffered a frameshift that restored an ancestral sequence.) Another 86 genes intact in MG1655 are missing altogether from ER2796, caused by 6 independent deletion events. Twenty-three genes in ER2796 are not present in MG1655 and were introduced primarily by insertion sequences. Finally, 109 genes in ER2796 suffered one or more missense mutations relative to their MG1655 orthologs. These mutations are a subset of the 249 SNPs identified relative to MG1655.

We similarly annotated a total of 174 RNA-coding genes in ER2796, including 89 tRNA, 22 rRNA, 2 tmRNA, and 61 ncRNA genes. Two RNA genes from MG1655 are missing from ER2796, *arcZ* (a ncRNA that acts as a positive antisense regulator of *rpoS*) and *symR* (a ncRNA that destabilizes the mRNA of *symE*, which is also missing from ER2796). Automated annotation of ER2796 also identified a cryptic tRNA gene at 347,232–347,312 that is also present but not annotated in MG1655; this is presumably a pseudogene and was not annotated in ER2796.

A complete list of all mutations relative to MG1655 is shown in S1 Table, and a complete list of all genes affected by these mutations is shown in S2 Table. Strain ER3413 was also completely sequenced, and in addition to the engineered disruption of *yhdJ*, we observed seven single base changes, one single base insertion, and a likely gene conversion event relative to ER2796 (S3 Table).

### Analysis of DNA methylation in ER2796

We used the program RS_Modification_and_Motif_Analysis.1 (Pacific Biosciences) to confirm the absence of methylation in the ER2796 sequence. As expected, no methylated motifs were identified at modification quality value (QV) thresholds of 30 or 20. To look for possible sparse methylation of ATGCAT sites indicative of M.EcoKII activity, we examined each of the 1644 instances of the site (on both strands) in the reference for possible methylation at the second A residue. Although 162 of these residues had mean IPD ratios with QV values greater than 20, none were identified by the program as having a characteristic m^6^A kinetic signature. In addition, we repeated the analysis using a negative control sequence, TTGCAA, and obtained a comparable result (166/2004 with QV > 20, compared to 162/1644). Thus, despite the presence of an intact *yhdJ* gene in this strain, there is no evidence of activity in the sample sequenced here.

### Mutations underlying the ER2796 genotype

The ER2796 genotype comprises 18 genetic markers with known phenotypes, including 15 historical markers based on its lineage and 3 additional markers revealed by sequencing (Table 3). These last 3 are changes from MG1655 found by others in various K-12 strains and shown to have phenotypic consequences: *rpoS396* [36], *luxS11* [37] and *rph* WT [38]. EcoGene [39], EcoCyc [40] and the resources of the *E*. *coli* Genetic Stock Center (CGSC) were relied on for gene, function and pedigree information. The newly found markers have subtle phenotypes but may have been selected by geneticists nonetheless, since stable markers in a healthy background are easier to work with. The wild type state of *rph* promotes growth in minimal media by improving pyrimidine biosynthesis, thus fostering a desirable healthy state. The *rpoS396* allele is at least partially suppressed by the accompanying amber suppressor [36]. At least one attempt to replace the suppressor in this lineage by transduction was foiled (EAR, unpublished observation), consistent with selective pressure to maintain suppression of *rpoS396*. The *luxS11* allele would interfere with "social" interactions mediated by quorum sensing [41], but this lineage has not been used to study this phenomenon.

**Table 3:** Genotype markers in ER2796 and underlying sequence features.

Allele	Old Allele Name (if changed)	Alteration	Genes Affected	ER2796 Sequence	MG1655 Sequence	Amino Acid Changes
*fhuA2*::*IS2*	*fhuA2*	*IS2* disruption	*fhuA* (b0150)	167920–169255 (169251–169255 is target site duplication)	between 167919–167920	ER2796_149 (aa 1–145 + 13 aa), and ER2796_151 (aa 158–747)
*ΔlacZ4826*	*Δ(lacZ)r1*	deletion	*lacZ* (b0344)	between 365092–365093	362419–364862	Δ223–1024; adds 40 aa extension overlapping *lacY*
*glnX44* ^a^	*glnV44*	tRNA transition	*glnX* (b0664)	693302 (T)	695693 (C)	(DNA nt) G34A
e*14* ^–^ (McrA^–^)	*mcr-62*	excision	*ymfDE*, *lit*, *intE*, *xisE*, *ymfIJ*, *cohE*, *croE*, *ymfLM*, *oweE*, *aaaE*, *ymfR*, *bee*, *jayE*, *ymfQ*, *stfP*, *tfaPE*, *stfE*, *pinE*, *mcrA* (b1137-b1141, b1143-b1148, b4692-b4693, b1150-b1159, respectively)	between 1193386–1193387	1195598–1210801	null; associated changes in *icd* sequence
*trpE31*	*trp-31*	missense	*trpE* (b1264)	1302017 (T)	1319610 (C)	G454D (ER2796_1284)
*dcm-6*		silent	*dcm* (b1961)	2012149 (T)	2029184 (C)	E386
		nonsense (TGA)^b^	*dcm* (b1961)	2013172 (T)	2030207 (C)	W45stop (ER2796_2013; also ER2796_2012 from internal start at aa 111)
*yedZ501*::*Tn10*(Tet^R^)	*zed-501*::*Tn10*	*Tn10* insertion	*yedZ* (b1972)	2021730–2030885 (2030877–2030885 is target site duplication)	between 2038764–2038765	Δ87–211; ER2796_2025 (aa 1–86 + 15 aa), and ER2796_2035 (from internal start at aa 102)
*Δ(hisG)1*	*hisG1*(Fs)	deletion, in-frame	*hisG* (b2019)	between 2080789–2080790	2088669–2088704	Δ152–163 (ER2796_2082)
*luxS11* ^c^		–1 frameshift	*luxS* (b2687)	between 2798558–2798559	2812480 (A)	Δ92–171; ER2796_2763 (aa 1–90 + 20 aa), and ER2796_2762 (from internal start at aa 108)
		silent	*luxS* (b2687)	2798561 (C)	2812483 (T)	L91 (ER2796_2762)
*rpoS396*(Am)^d^		nonsense (TAG)	*rpoS* (b2741)	2851555 (A)	2865477 (G)	E33stop ER2796_2821 (from internal start at aa 40)
*argG6*(Fs)	*argG6*	–1 frameshift	*argG* (b3172)	between 3304561–3304562	3317286 (C)	Δ210–447 ER2796_3265 (aa 1–209 + 13 aa), and ER2796_3266 (from internal start at aa 221)
*rpsL104*		missense	*rpsL* (b3342)	3443238 (G)	3472313 (T)	K88Q
		missense	*rpsL* (b3342)	3443372 (G)	3472447 (T)	K43T (ER2796_3428)
*Δdam-16*::*Kan* ^*R*^		deletion + 1266 bp Kan^R^ insertion	*dam* (b3387)	3484166–3485431	3513241–3513773	Δ55–242 ER2796_3474 (from internal start at aa 242)
*xyl-7*		missense	*xylB* (b3564)	3699368 (A)	3726511 (G)	A295V (ER2796_3669)
		missense	*xylA* (b3565)	3700835 (A)	3727978 (G)	H271Y (ER2796_3670)
		silent	*xylA* (b3565)	3700836 (G)	3727979 (A)	N270
		insertion (IS1)	*xylF* (b3566)	3702309–3703085	between 3729451–3729452	Δ100–330; adds 1 aa extension (ER2796_3671)
*mtlA2*(Fs)	*mtlA2*	–2 frameshift	*mtlA* (b3599)	between 3744696–3744697	3771063–3771064 (GG)	Δ254–637 ER2796_3708 (aa 1–253 + 60 aa), and ER2796_3709 (from internal start at aa 306)
*rph* ^WT^ ^e^		+1 frameshift	*rph* (b3643)	3787535 (C)	between 3813902–3813903	ER2796_3754
*metB1*(Fs)	*metB1*	–2 frameshift	*metB* (b3939)	between 4100468–4100469	4126836–4126837 (CG)	Δ48–386; ER2796_4061 (8 aa + aa 48–386)
*Δ(fimB-opgB)114*::*IS10*(RM^–^)^f^	*Δ(mcr-hsd-mrr)114*::*IS10*	deletion of 60,679 bp + insertion of 2 *IS10* elements in direct repeat	*fimBEAICDFGH*, *gntP*, *uxuABR*, *yjiC*, *iraD*, *yjiE*, *iadA*, *yjiGH*, *kptA*, *yjiJKLMN*, *mdtM*, *yjiPRSTV*, *mcrCB*, *symER*, *hsdSMR*, *mrr*, *yjiAXY*, *tsr*, *yjjLMN*, *opgB* (b4312-b4337, b4339-b4342, b4486, b4345-b4347, b4625 [ncRNA], b4348-b4359, respectively)	4511776–4514442 (4511776–4513104 and 4513114–4514442 are *IS10*, 4513105–4513113 is target site duplication [inverted])	4537567–4595455	null, except *opgB* (b4359) Δ671–763; ER2796_4466 (aa 1–670)

^a^ The reassignment of *glnV44* (*supE44*) was noted previously [43].

^b^ The double mutation (one silent) is in agreement with a previous study [35].

^c^ The sequence of *luxS* reported here is identical to a previous study [70], although our alignment differs slightly, moving the frameshift 3 nt and inferring a transition instead of a transversion. The steps that resulted in the shared *luxS11* allele clearly include a base deletion and a base change, but exactly which deletion and which base change depend on the local alignment. Spontaneous unselected transitions are somewhat more frequent than transversions [71], so our alignment may be preferable. The mutation is present in DH1 [72] (see Table 1), an ancestor of the strain used in [70] and may have been present in sibling strains JC1552 (ancestral to ER2796; RecA^+^) and JC1553 (source of the *recA1* allele of the DH1 and its descendants [73, 74]). The *luxS* and *recA* genes are very close, about 8 kb apart, and introduction of *recA1* was the last step in construction of DH1.

^d^ This nonsense mutation, which is common in laboratory *E*. *coli* strains [75], was most likely ancestral, not introduced by transduction. It may be partially suppressed in this strain. The *rpoS* mutation and the accompanying *supE44* mutation (identified here as *glnX44*) can be traced to strain Y10, very early in the K-12 pedigree [28].

^e^ This frameshift mutation presumably restores the wild type state, reverting the frameshift present in early K-12 derivative strains MG1655 and W3110 [76].

^f^ The position of the parental *zjj202*::*Tn10* is inferred to be 4597466–4597474 of MG1655 (NC_000913.2), the nine base target sequence that is duplicated upon insertion.

Of the 15 purposely-introduced markers, the nine found in JC1552 (Fig 2) were generated during early studies of genetic processes, and six more were introduced during more recent studies of genetic processes. These 15 exhibit properties desirable for genetic study: they have strong phenotypes and are stable. 13 of the 15 are indels or nonsense mutations.

Of the full set of 18 markers, three had already been characterized at the sequence level prior to this study. Single-base changes led to the *glnX44* amber suppressor (formerly attributed to the adjacent duplicate tRNA *glnV*) [42, 43], and the *dcm-6* opal and silent mutations [35] (although we did not observe the Q26R mutation described there). As expected, the *mcr-62* allele is an excision of the e*14* prophage. Its sequence is identical to that of DH10B, which carries an independent excision allele in a different lineage [4]. Excision is a relatively frequent event that restores the sequence of *icd* to a presumptively ancestral state by fusion of the N-terminus of *icd* to the original C-terminus, the "pseudogene" *icdC* [44]. We rename this allele "e*14* ^–^ (McrA^–^)" to match other strains.

The remaining markers from the Bachmann pedigree [28] include an IS2 insertion (*fhuA2*::*IS2*), three frameshifts [*argG6*(Fs), *mtlA2*(Fs) and *metB1*(Fs)], a double missense (*rpsL104*) and a single missense (*trpE31*). The *xyl-7* allele includes four mutations in three of the six genes of the regulon: a conservative missense change in *xylB*, a nonconservative and a silent change in *xylA*, and an IS1 insertion in *xylF*. The *Δ(hisG)1* mutation is unexpectedly found to be a 36 nt deletion, rather than a frameshift mutation. This deletion removes a sequence flanked by four-base direct repeats of ACTG, and one of the repeats. Isolation of a more stable allele during lineage manipulation may account for the conflict with the original report of a revertible allele [36]. Others have reported to CGSC that this allele is non-revertible (John Wertz, personal communication).

**Fig 4:**
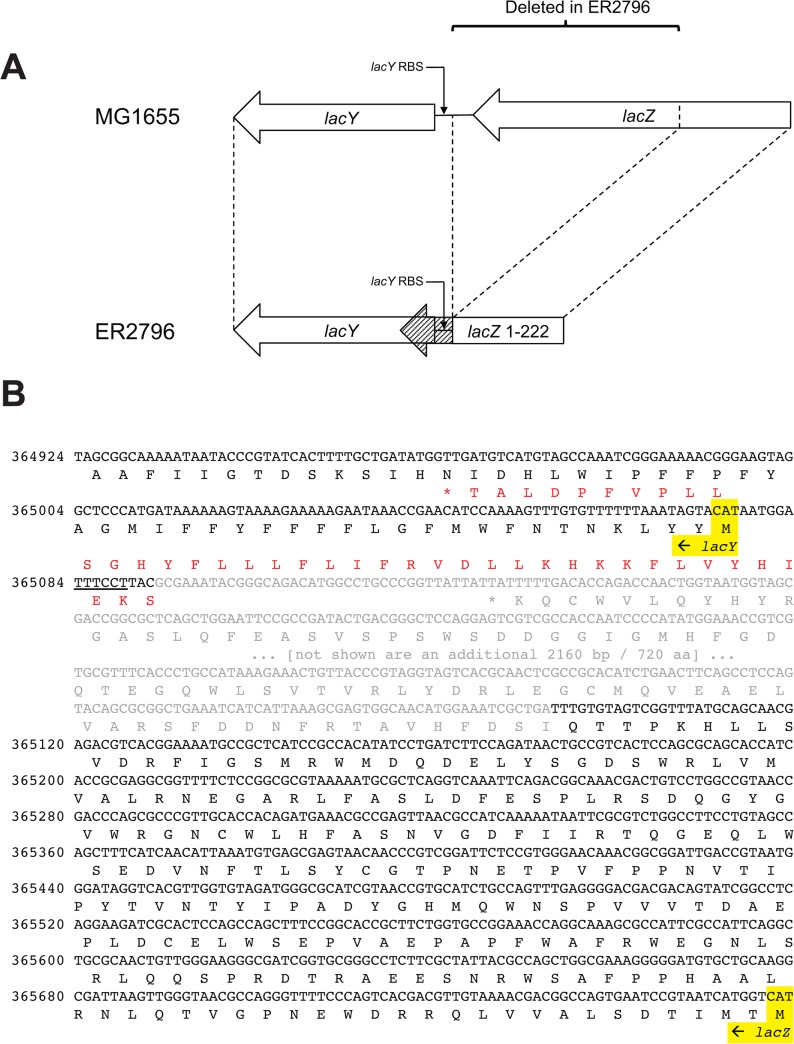
Comparison of the *lacZY* regions of MG1655 and ER2796. A. Schematic drawing showing the region of MG1655 *lacZ* and *lacZY* intergenic region that is deleted in ER2796. It is oriented forward with respect to the chromosomal sequence, with the operon reversed from the conventional representation. In ER2796, the *lacZ* ORF enodes amino acids 1–222 of MG1655 *lacZ* (white box) fused to 40 amino acids derived from the *lacZY* intergenic region, and overlapping with *lacY* (cross-hatched box). The putative *lacY* ribosome binding site (RBS) is preserved in ER2796. B. DNA and translated protein sequence of the *lacZY* junction, numbered from ER2796. Nucleotides and translated amino acids missing in ER2796 are shown in gray, and those present are shown in black. In ER2796, aa 1–222 of the translated ORF are shown in black, and the 40 aa derived from the intergenic region are shown in red. Start codons of *lacZ* and *lacY* are highlighted, and the putative RBS of *lacY* is underlined. 2160 bp (720 aa) of MG1655 *lacZ* have been removed at the indicated position for brevity.

The *ΔlacZ4826* (formerly *Δ(lacZ)r1*) marker is a large *lacZ* deletion that permits LacYA activity. Historically, it was used to characterize the phenomenon of transcriptional polarity [45, 46] in which long untranslated regions in mRNA result in RNA degradation and reduced expression of genes later in in the operon. By bringing the *lacY* translation initiation site close to nonsense mutations early in *lacZ*, it restored much of the activity of *lacYA* that was lost due to early translation termination in the single nonsense mutants. Here we find that the deletion border is actually within the *lacZ-lacY* intergenic region (Fig 4A). The *lacZ* fragment encoding the N-terminal region is extended by 40 codons overlapping the *lacY* start codon (Fig 4B). The LacY activity is weak in the single mutant configuration (determined from growth on melibiose at 42°C) [47], suggesting that translation of the LacZ chimeric protein may compete with LacY initiation when translation is not stopped early.

The remaining three markers were engineered (*dam-16*::*Kan* ^*R*^) [48], or transposon-mediated [*yedZ501*::*Tn10* [49] and *Δ(fimB-opgB)114*::*IS10*(RM^–^) [31]]. *Δ(fimB-opgB)114*::*IS10* was mediated by the parental *zjj202*::*Tn10*, which rearranged to remove the unique Tn10 material and adjacent DNA. Deletion of the highly variable immigration control region (ICR) was selected for, yielding an inversion/deletion event that removed adjacent DNA and left a tandem IS10 repeat at the position of the original insertion. The positions of the *yedZ501*::*Tn10* and the distal IS10 from *zjj202* agree with those reported by Nichols [50].

### Gene conversion events

We found four instances of clustered SNPs (relative to MG1655) in repeat loci for which a perfect donor copy at a different locus could be identified. These properties strongly suggest gene conversion events. The unidirectional information transfer process can occur by multiple mechanisms [51, 52].

**Fig 5:**
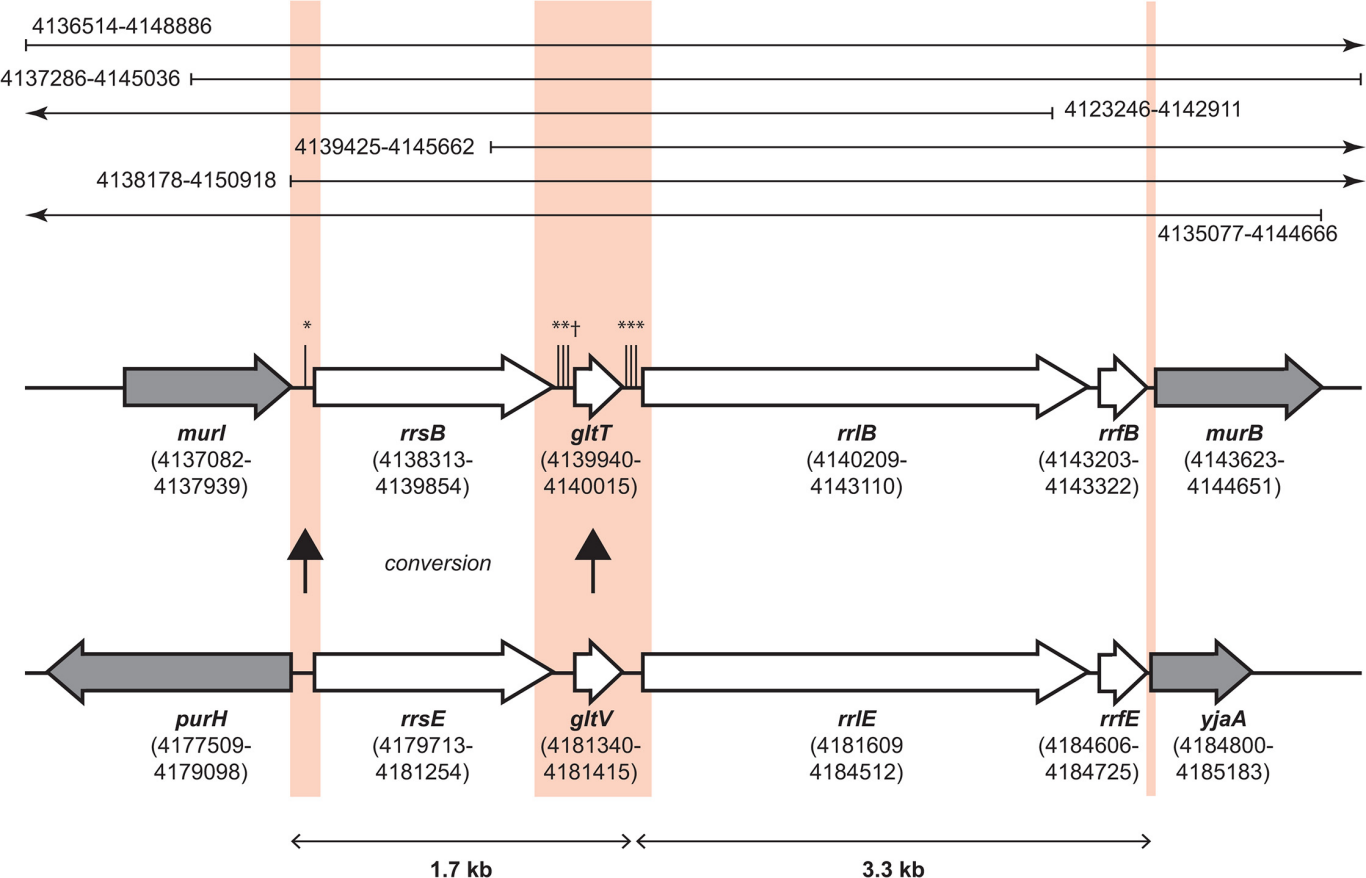
Use of long reads to identify gene conversion events. The schematic alignment shows the paralogous ribosomal gene clusters *rrnB* and *rrnE* from ER2796 (white genes) along with nonhomologous flanking genes (gray). The genes are marked with names and coordinates in ER2796. In ER2796, *rrnB* has been the apparent recipient of a gene conversion event in which *rrnE* served as donor (vertical arrows), and thus both regions are identical. As a result of this event, *rrnB* in ER2796 exhibits minor variations when compared with *rrnB* from its ancestor, MG1655: six SNPs (marked with *) and one indel (marked with †). Red tinted boxes indicate the regions of alteration (left and middle) and delineate the boundaries of the clusters (left and right). Sequencing reads internal to the clusters (i.e., between the outer two red boxes) cannot be mapped uniquely to one locus or the other unless they extend into the nonhomologous flanking regions, and the minor variants within (e.g., the middle red box) cannot be assigned to one cluster or the other without sequencing reads directly connecting them with a flanking region on one side or the other. The long-read library used in this analysis includes numerous reads that connect the unique flanking regions with the internal variants. The mapped coordinates of six example reads from the actual analysis are shown at the top, including some that span both sides of the 5 kb gene cluster. Arrows indicate where a read continues beyond the region shown here.

Three of the seven rRNA operons have served as information recipients; in one case a unique donor was identified. The most compelling includes the entire *rrsB* gene and the intergenic region between *rrsB* and *rrlB*. Six SNPs, a deletion of 20 bp and an insertion of 106 bp span coordinates 4,138,303–4,140,042 (corresponding to MG1655 4,166,238–4,166,499) (S1 Table). The sequence aligns perfectly with the corresponding sequence of *rrsE-rrlE*, both in ER2796 and in MG1655 (Fig 5). The contiguous clustering of the six base changes, a 106 bp deletion and a 20 bp insertion strongly suggest unidirectional information transfer from *rrsE-rrlE* into *rrsB-rrlB*, i.e. gene conversion, rather than 8 separate mutational events, even if a ready mechanism were available to explain the insertion and deletion.

Two other cases involve clustered changes in *rrl* loci, although more than one donor locus is possible: 4 SNPs in *rrlG* are candidates for a conversion patch from one of four possible donors (*rrlA*, *C*, *E or H*), and 3 SNPs in *rrlD* are candidates for a conversion patch from any of the other six *rrl* loci. This conversion patch is shared with DH10B.

Authentic SNPs do occur in rRNA genes however. No candidate donor was found for two SNPs in *rrlC* in any of 6 K-12 genome sequences at NCBI. All 42 hits (7 operons per genome) had two mismatches at the same positions.

In addition to the ribosomal operons, a fourth gene conversion patch was observed between two prophage sequences. Eight SNPs in the Qin prophage represent conversion to identity with a similar sequence in the Rac prophage. The conversion makes the *ydfK* transcript identical to that of *ynaE*. The *ydfK* upstream untranslated region (UTR) acts as an RNA-mediated thermosensor, and the *ynaE* UTR was inferred to function similarly, from similarity of predicted secondary structure [53].

### Indels

There are 42 insertions and deletions in ER2796 compared to MG1655 (S1 Table). Of these, 18 are 1–2 bp in length, resulting in frameshifts in 13 protein-coding genes; 14 are simple gain or loss of mobile elements (discussed below); one is a 16 kb deletion promoted by IS recombination; 4 are selected or engineered deletions (discussed above); and 5 are larger intergenic indels ranging from 8–181 bp in length. Besides the 13 frameshifted protein-coding genes, another has an in-frame deletion, 9 are disrupted by mobile elements or engineered deletions, and 2 RNA genes have 1–2 bp indels, for a total of 25 genes. Finally, large-scale deletions resulted in the loss of 86 genes relative to MG1655, primarily in prophages: 5 in the loss of DLP12, 18 by the loss of e14, 2 by the loss of IS1H at the *flhD* locus, 9 by the loss of CPZ-55, 9 by the IS5R-promoted deletion, and 43 by the loss of the ICR region. Aside from IS sequences, the only genes gained by ER2796 relative to MG1655 are *aph* (ER2796_3475), encoding kanamycin resistance (a consequence of *dam* inactivation), and the 7 unique genes of Tn10, including *tetA* (ER2796_2029), encoding tetracycline resistance.

### Polymorphisms

ER2796 contains a total of 249 single-base changes relative to MG1655 (S1 Table), including the 21 that we propose result from 4 gene conversion events (discussed above). These result in 116 missense mutations, 9 nonsense mutations, and 68 silent mutations in protein-coding genes, 11 changes in RNA-coding genes, and 38 base changes in intergenic regions. One of the missense mutations alters a former stop codon to allow readthrough. The nonsense mutations result in known inactivation of the products of *dcm* and *rpoS* (Table 3), and truncation of the products of *rssB*, *ydbH*, *htpX*, *rsmF*, *hycC*, *cptB*, and *galP* (S1 and S2 Tables). The missense mutations occur in a total of 110 different genes, and between missense, nonsense, and RNA-coding mutations, a total of 124 gene products are altered through polymorphism relative to MG1655 where they occur in both genomes.

By contrast, DH10B contained a total of 132 single-base changes relative to MG1655, resulting in 66 genes with missense and 5 with nonsense mutations [4]. A total of 17 SNPs are shared between ER2796 and DH10B, some of which may have been acquired in the DH10B lineage through a genetic cross with W677, an ancestor of ER2796, or through other crosses. Although it might be suggested that the greater number of SNPs in ER2796 is due to the mutator phenotype resulting from *dam* inactivation, that mutation was introduced only recently in the ER2796 lineage (Fig 1), and ER2796 and DH10B have comparable numbers of SNPs in intergenic regions (38 and 42, respectively).

### Mobile elements

Many of the larger indels in ER2796 discussed above result from the gain or loss of mobile elements relative to MG1655. These include four IS1 insertions and one associated deletion, two IS5 insertions and one associated deletion, one IS2 insertion, and two solo IS10 insertions in addition to the four IS10s associated with deliberately introduced changes (discussed above).

The lack of an IS element (IS1H in the case of MG1655) at the regulatory region of the *flhD* operon may lead to a poor motility phenotype in ER2796. The *flhD* operon is the master operon of the flagellar regulon, and the presence of an IS element there has been shown to increase operon expression and is associated with high motility [54]. Of the genes disrupted by IS insertions, *nohD* is a part of the DLP12 prophage not otherwise lost; *xylF* and *fhuA* are part of known alleles (Table 3); *mglA* is part of the beta-methylgalactoside ATP-Binding Cassette transporter, and its inactivation is expected to impair galactoside uptake [55]; *tdcD* participates in threonine degradation; and *rclA* is essential for survival of reactive chlorine stress [56].

Three MG1655 prophages are missing or compromised in ER2796: DLP12 (3.4 kb, partial loss associated with a new IS1A insertion), CPZ-55 (6.8 kb, precise excision), and e*14* (16 kb, precise excision). The largest deletion, 55 kb, was mediated by the parental *zjj202*::*Tn10* insertion, discussed above. The genes lost in these four major events primarily consist of phage-related functions, but the removal of e*14* and the ICR results in the loss of all of the restriction-modification systems from MG1655, namely *mcrA*, *mcrBC*, *mrr*, and *hsdRMS* (EcoKI). In all, 83 genes were completely deleted in these events, and 3 additional genes (*ybcV*, *eutA*, and *opgB*) were disrupted.

### Growth properties

Growth curves were determined for MG1655 and ER2796 in Rich medium, and exponential growth rate constants were calculated as 0.0272 for MG1655 and 0.0197 for ER2796. These correspond to doubling times of approximately 25 min for MG1655 and 35 min for ER2796.

## Discussion

### Gene conversion events

Examination of the complete sequence of ER2796 revealed the occurrence of four gene conversion events between repeated sequences. Gene conversion is a nonreciprocal recombination process in which one copy of a diverged repeat donates its sequence to another, erasing the recipient version. These are evolutionarily important events that have rarely been confirmed in bacterial systems [57, 58]. Intragenomic gene conversion can counter mutational drift of repeated sequences, homogenizing them, or can be programmed to generate variation, as when silent-locus copies are donated to expression loci in antigenic phase variation (e.g., [59]). Here, gene conversion would contribute to the bewildering network of phage interrelationships [60]. The fact that ER2796 has a known pedigree with no horizontal transfer from outside the lineage, together with the long reads enabled by the Pacific Biosystems SMRT sequencing platform, enabled detection of these events. Detection depends on the presence of divergent copies in parent participants, on a complete inventory of parental copies and of the resulting offspring (i.e., complete genomes), and crucially, on correct assembly of long repeats (>5 kb) such as ribosomal operons (Fig 5).

### Utility of MTase-deficient strains

The availability of strains of *E*. *coli* that are completely defective in DNA methylation has obvious advantages for studying the methylation specificity of cloned DNA MTase genes. This has already been realized in a number of studies [25, 26, 61, 62] and will be extremely useful going forward when confirmation of inferences made by whole genome sequencing using SMRT technology need to be confirmed. The absence of the Type I R-M system (EcoKI) means that this strain is more easily transformable since EcoKI is the only known ENase in *E*. *coli* K-12 recognizing unmethylated DNA. Similarly, the absence of the methylation dependent restriction ENases (McrA, McrBC and Mrr) mean that this strain is also suitable for cloning intact restriction systems that may otherwise be restricted because of the introduced MTase.

It should be noted that the loss of the Dam MTase confers a mutator phenotype on ER2796 and ER3413. Consequently, for SMRT sequencing analysis of the activities of cloned MTases, we perform all plasmid construction and propagation steps in other (Dam^+^) strains of *E*. *coli*, utilizing ER2796 or ER3413 only at the final step, namely isolation of total or plasmid DNA for sequencing and methylation analysis. In addition, we routinely check the sequencing reads against the MTase gene as reference to ensure the absence of introduced mutations. In our experience, such mutations are rare. In any case, given the large number of DNA MTases that recognize GATC both in bacterial, archaeal and phage genomes, a strict test of their specificity can only be conducted by cloning the genes into one of these two strains. Already a number of DNA MTases recognizing GATC have been successfully characterized using ER2796 and subtle variations in specificity involving the selectivity for flanking nucleotides have been successfully detected. We anticipate that these two strains will be invaluable as further interest develops in the methylation patterns that provide the epigenetic marks on bacterial and archaeal DNAs.

## Supporting Information

S1 Fig (A) Detection of G^m6^ATC methylation status by methylation protection assay with MboI and DpnI, and ATGC^m6^AT by NsiI, in gDNAs from 2796 (lanes 1), 2796 [pUC19:MEcoKIIwt] (lanes 2), 2796 [pUC19:MEcoKIIΔAgeI] (lanes 3) and S17-1 [pRE112:MEcoKIIΔAgeI] (lanes 4).
(B) Colony PCR screening for the site-specific recombination event at the *yhdJ* locus in the first round of recombination (clones 9 and 18) and second round of recombination (clones 9–3, 9–7, 9–8, 18–3, 18–4, 18–6, 18–7, 18–8, 18–13, and 18–15). (C) Identification of wild type and mutant alleles of *yhdJ* by AgeI restriction digestion of colony PCR fragments.
(PDF)

S1 Table List of sequence changes from the reference strain MG1655 to ER2796. (A) Insertions and deletions. (B) SNPs and substitutions.
(PDF)

S2 Table List of ORFs with nonsynonymous changes from the reference strain MG1655 to ER2796. (A) Disrupted ORFs. (B) ORFs with missense mutations.
(PDF)

S3 Table List of additional sequence changes from the reference strain MG1655 to ER3413 over and above those in ER2796. (PDF)
