# Supplementary figures and images for: Complete Genome Sequence of ER2796, a DNA Methyltransferase-Deficient Strain of Escherichia coli K-12
Source: PLoS One. 2015 May 26;10(5):e0127446. doi: 10.1371/journal.pone.0127446 (PMC4444293; doi:10.1371/journal.pone.0127446)

**Figure S1**

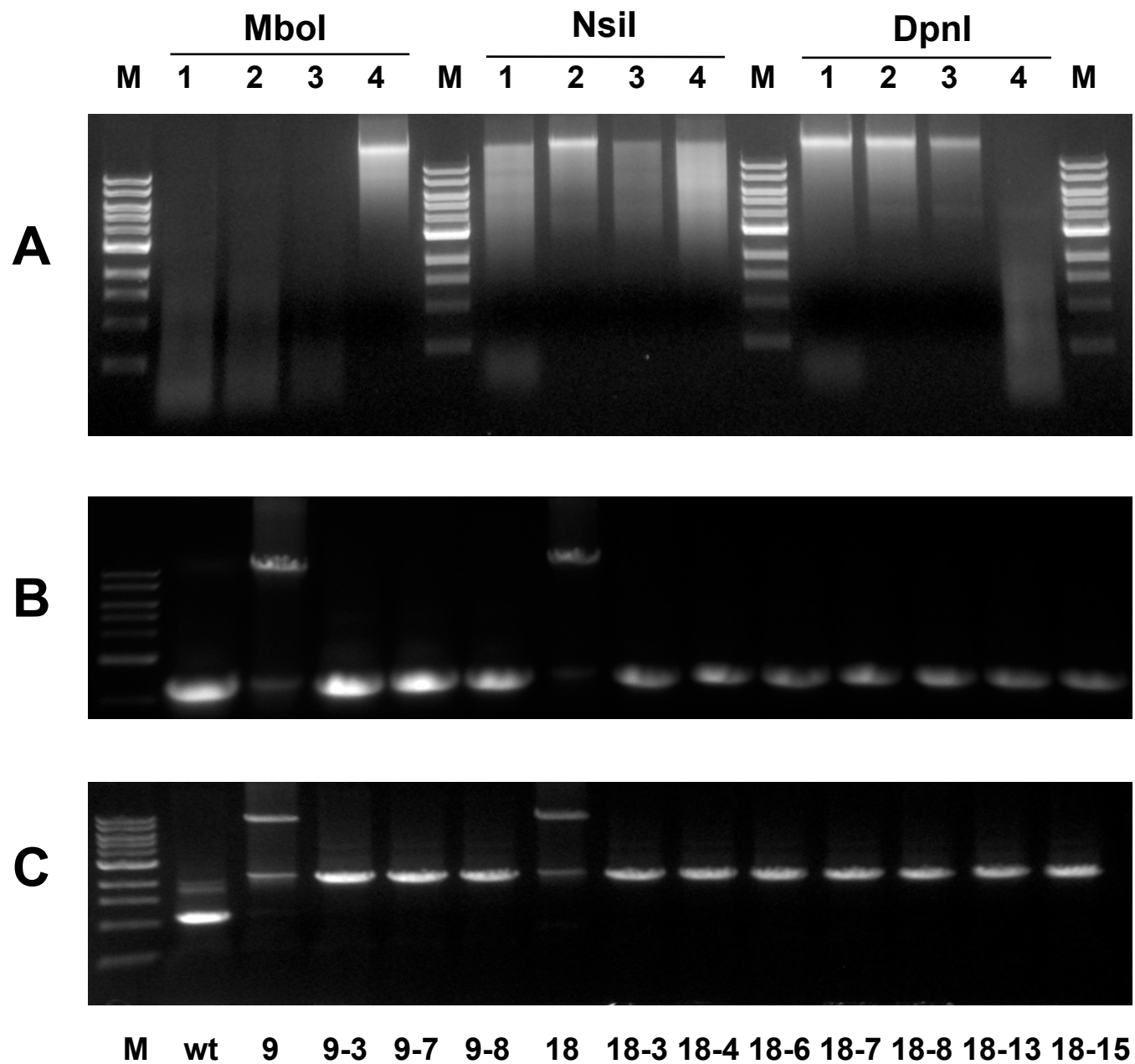

Supplement: S1 Fig — (A) Detection of Gm6ATC methylation status by methylation protection assay with MboI and DpnI, and ATGCm6AT by NsiI, in gDNAs from 2796 (lanes 1), 2796 [pUC19:MEcoKIIwt] (lanes 2), 2796 [pUC19:MEcoKIIΔAgeI] (lanes 3) and S17-1 [pRE112:MEcoKIIΔAgeI] (lanes 4). (B) Colony PCR screening for the site-specific recombination event at the yhdJ locus in the first round of recombination (clones 9 and 18) and second round of recombination (clones 9–3, 9–7, 9–8, 18–3, 18–4, 18–6, 18–7, 18–8, 18–13, and 18–15). (C) Identification of wild type and mutant alleles of yhdJ by AgeI restriction digestion of colony PCR fragments. (PDF) [file pone.0127446.s001.pdf]
