# Supplementary material for: Complete Genome Sequence of ER2796, a DNA Methyltransferase-Deficient Strain of Escherichia coli K-12
Source: PLoS One. 2015 May 26;10(5):e0127446. doi: 10.1371/journal.pone.0127446 (PMC4444293; doi:10.1371/journal.pone.0127446)
Supplement: S1 Table — (A) Insertions and deletions. (B) SNPs and substitutions. (PDF) [file pone.0127446.s002.pdf]

Table S1A. List of sequence changes from the reference strain MG1655 to ER2796 (insertions and deletions).

Type	MG1655	ER2796	Length (bp)	Description or Consequence	Footnotes
insertion	bet 167919-167920	167920-169255	1336	fhuA::IS2	
insertion	bet 317993-317994	319330-320667	1338	rclA::IS10	
deletion	362419-364862	bet 365092-365093	2444	lacZ Δ223-1024, adds 40 aa extension overlapping lacY	
deletion	379237-379237	bet 379466-379467	1	intergenic fs, between frmR-yaiO	a
insertion	bet 547831-547832	548061-548061	1	ylbE +1 fs	b
deletion	575013-578410	bet 575242-575243	3398	DLP12 prophage deletion (nmpC-borD)	
insertion	bet 580216-580217	577049-577825	777	nohD::IS1	
deletion	872620-872620	bet 870228-870229	1	yliE –1 fs	
insertion	bet 1096728-1096729	1094337-1094517	181	intergenic fs, between ycdU-serX	
deletion	1195598-1210801	bet 1193386-1193387	15204	e14 prophage deletion (ymfD-mcrA)	
deletion	1286098-1286275	bet 1268682-1268683	178	intergenic deletion, between narI-rttR	
deletion	1341944-1341944	bet 1324350-1324351	1	yciT –1 fs	
deletion	1423350-1423350	bet 1405755-1405756	1	intergenic fs, between trkG-ynaK (rac prophage)	
deletion	1553754-1553754	bet 1536158-1536159	1	intergenic fs, between maeA-sra	
insertion	bet 1635044-1635045	1617449-1618786	1338	IS10 in intergenic ynfO-ydfO	
deletion	1791400-1791400	bet 1775141-1775142	1	btuD –1 fs	
deletion	1976527-1977302	bet 1960267-1960268	776	loss of IS1H from flhD operon [Barker et al 2004, J Bacteriol]	
insertion	bet 2038764-2038765	2021730-2030885	9156	yedZ::Tn10	
deletion	2088669-2088704	bet 2080789-2080790	36	hisG Δ152-163, in-frame deletion	
deletion	2109280-2109280	bet 2102364-2102365	1	rfbD –1 fs	
insertion	bet 2235901-2235902	2227986-2228762	777	mglA::IS1	
deletion	2358572-2358572	bet 2351432-2351433	1	rhmD –1 fs	
insertion	bet 2471415-2471416	2464276-2464276	1	oweS (pseudogene) +1 fs	
deletion	2556719-2563508	bet 2549579-2549580	6790	CPZ-55 prophage deletion	
insertion	bet 2763433-2763434	2749505-2749512	8	intergenic insertion, between yfjL-yfjM (CP4-57 prophage)	a
deletion	2812480-2812480	2798558-2798559	1	luxS –1 fs	
deletion	3205570-3205570	3191647-3191648	1	ttdB –1 fs	
insertion	bet 3260834-3260835	3246953-3248151	1199	tdcD::IS5	
deletion	3317285-3317285	bet 3304560-3304561	1	argG –1 fs	
deletion	3347229-3363578	bet 3334503-3334504	16350	Δ(mtgA-yhcE), promoted by IS5R	
net insertion	3513241-3513773	3484166-3485431	733	dam Δ55-242, 533 bp deletion, 1266 bp insertion (KanR)	
insertion	bet 3694452-3694453	3666111-3667309	1199	IS5 in intergenic yhjR-bcsE	

insertion	bet 3729451-3729452	3702309-3703085	777	xylF::IS1	
deletion	3771063-3771064	bet 3744696-3744697	2	mtlA –2 fs	
insertion	bet 3813902-3813903	3787535-3787535	1	rph +1 fs	
deletion	4126834-4126835	bet 4100466-4100467	2	metB –2 fs	
net deletion	4166289-4166394	4139920-4139939	86	intergenic 106 bp deletion, 20 bp insertion, between rrsB-gltT	c
deletion	4169461-4169462	bet 4143005-4143006	2	rrlB (rRNA) 2 nt deletion	
insertion	bet 4233537-4233538	4207081-4207857	777	IS1 in intergenic pgi-yjbE	
deletion	4294293-4294403	bet 4268612-4268613	111	intergenic deletion in RIP321 repeat region	
net deletion	4537567-4595455	4511776-4514442	55222	Δ(fimB-opgB)::IS10+IS10; 57,889 bp deletion, 2667 bp insertion	
insertion	bet 4604109-4604110	4523097-4523097	1	leuV (tRNA) 1 nt insertion	a

Footnotes

a	Not in ER3413
b	Not a real change; sequence error in MG1655 file NC_000913.2, corrected in NC_000913.3; ylbE not listed in Table S4 for this reason
c	From the same gene conversion event responsible for the block of changes at MG1655 4164671-4166499 in the SNP table

Table S1B. List of sequence changes from the reference strain MG1655 to ER2796 (SNPs and substitutions).

Type	MG1655	ER2796	Change	Gene	Locus	Codon Change	Type	AA Change	ER2796 ORF	Footnotes
SNP (transition)	10,581	10581	C -> T	–	–	–	INTERGENIC	–	–	
SNP (transition)	20,756	20756	G -> A	–	–	–	INTERGENIC	–	–	
SNP (transition)	21,047	21047	G -> A	rpsT	b0023	GCC -> GTC	MISSENSE	A11V	ER2796_18	
SNP (transition)	23,918	23918	G -> A	ileS	b0026	GAT -> AAT	MISSENSE	D510N	ER2796_20	
SNP (transition)	32,355	32355	C -> T	carB	b0033	ATC -> ATT	SILENT	I513	ER2796_28	
SNP (transition)	73,439	73439	C -> T	thiP	b0067	GCG -> GCA	SILENT	A361	ER2796_62	
SNP (transition)	94,278	94278	G -> A	murE	b0085	GCG -> GCA	SILENT	A371	ER2796_82	a
SNP (transversion)	134,256	134256	G -> T	–	–	–	INTERGENIC	–	–	b
SNP (transition)	156,068	156068	A -> G	ecpD	b0140	GTA -> GCA	MISSENSE	V45A	ER2796_137	
SNP (transition)	157,043	157043	G -> A	–	–	–	INTERGENIC	–	–	
SNP (transition)	244,459	245795	G -> A	yafJ	b0223	GGC -> AGC	MISSENSE	G45S	ER2796_228	
SNP (transversion)	283,759	285095	T -> G	yagF	b0269	GCT -> GCG	SILENT	A445	ER2796_275	a
SNP (transition)	286,580	287916	C -> T	yagH	b0271	CTG -> TTG	SILENT	L190	ER2796_277	
SNP (transition)	288,315	289651	A -> G	yagI	b0272	CTT -> CTC	SILENT	L24	ER2796_278	
Substitution	305436-305437	306772-306773	GG -> AA	ecpD	b0290	TCC -> TTT	MISSENSE	S202F	ER2796_297	
SNP (transition)	314,745	316081	G -> A	insE1	b0298	CTG -> CTA	SILENT	L77	ER2796_305	
SNP (transition)	321,288	323962	C -> T	ykgE	b0306	CTG -> TTG	SILENT	L153	ER2796_314	
SNP (transition)	360,258	362932	G -> A	cynX	b0341	GGC -> GAC	MISSENSE	G348D	ER2796_349	
SNP (transition)	457,412	457641	G -> A	clpX	b0438	GAT -> AAT	MISSENSE	D255N	ER2796_448	
SNP (transition)	458,656	458885	C -> T	lon	b0439	TCT -> TTT	MISSENSE	S182F	ER2796_449	
SNP (transversion)	461,618	461847	C -> A	ppiD	b0441	CTC -> CTA	SILENT	L160	ER2796_451	
SNP (transition)	536,755	536984	C -> T	–	–	–	INTERGENIC	–	–	
SNP (transition)	542,487	542716	A -> G	ylbA	b0515	TAA -> CAA	READTHROUGH	stop262Q	ER2796_524	Adds aa 262-267
SNP (transversion)	542,488	542717	A -> T	ylbA	b0515	ATT -> ATA	SILENT	I261	ER2796_524	
SNP (transition)	547,694	547923	A -> G	ylbE	b4572	GAA -> GAG	SILENT	E38	ER2796_528	
SNP (transition)	627,792	625401	A -> G	entA	b0596	AAT -> GAT	MISSENSE	N7D	ER2796_607	
SNP (transition)	658,740	656349	G -> A	lipA	b0628	CTG -> TTG	SILENT	L234	ER2796_642	
SNP (transversion)	658,750	656359	G -> T	lipA	b0628	ACC -> ACA	SILENT	T230	ER2796_642	
SNP (transition)	661,214	658823	G -> A	lipB	b0630	CTC -> CTT	SILENT	L96	ER2796_644	
SNP (transition)	695,693	693302	C -> T	glnX	b0664	–	tRNA	–	–	
SNP (transition)	818,459	816068	C -> T	moaD	b0784	GAC -> GAT	SILENT	D63	ER2796_802	
SNP (transition)	855,657	853266	C -> T	ybiT	b0820	CCT -> TCT	MISSENSE	P158S	ER2796_838	
SNP (transversion)	856,264	853873	C -> A	ybiT	b0820	ACG -> AAG	MISSENSE	T360K	ER2796_838	
SNP (transition)	891,721	889329	G -> A	rimK	b0852	GAA -> AAA	MISSENSE	E178K	ER2796_870	
SNP (transition)	898,168	895776	G -> A	rlmC	b0859	CGT -> CAT	MISSENSE	R143H	ER2796_878	
SNP (transition)	908,317	905925	G -> A	ltaE	b0870	CTC -> CTT	SILENT	L67	ER2796_889	
SNP (transition)	961,728	959336	C -> T	rpsA	b0911	CGT -> TGT	MISSENSE	R171C	ER2796_932	
SNP (transition)	972,800	970408	C -> T	smtA	b0921	TCC -> TTC	MISSENSE	S14F	ER2796_942	

SNP (transversion)	1,004,914	1002522 T -> A	pyrD	b0945	ATT -> ATA	SILENT	I308	ER2796_968	
SNP (transition)	1,004,926	1002534 C -> T	pyrD	b0945	GCC -> GCT	SILENT	A312	ER2796_968	
SNP (transition)	1,005,312	1002920 C -> T	zapC	b0946	CCC -> CCT	SILENT	P46	ER2796_970	
SNP (transition)	1,010,843	1008451 G -> A	uup	b0949	GAA -> AAA	MISSENSE	E553K	ER2796_972	
SNP (transition)	1,042,830	1040438 G -> A	etk	b0981	CTG -> TTG	SILENT	L202	ER2796_1004	
SNP (transition)	1,049,315	1046923 C -> T	insA	b4516	TCA -> TTA	MISSENSE	S87L	ER2796_4627	c
SNP (transition)	1,049,315	1046923 C -> T	insB1	b0988	CTC -> CTT	SILENT	L22	ER2796_0911	c
SNP (transversion)	1,145,302	1143091 C -> A	yceF	b1087	GTT -> TTT	MISSENSE	V173F	ER2796_1115	
SNP (transversion)	1,157,125	1154914 G -> T	ptsG	b1101	GTC -> TTC	MISSENSE	V12F	ER2796_1130	
SNP (transition)	1,169,059	1166848 A -> G	ycfS	b1113	CTG -> CCG	MISSENSE	L180P	ER2796_1142	a
SNP (transition)	1,189,104	1186893 G -> A	phoP	b1130	ACC -> ACT	SILENT	T189	ER2796_1160	
SNP (transition)	1,189,203	1186992 A -> G	phoP	b1130	ACT -> ACC	SILENT	T156	ER2796_1160	a
SNP (transition)	1,219,716	1202301 C -> T	ycgH	b4491	TCC -> TTC	MISSENSE	S298F	–	d
SNP (transition)	1,232,892	1215477 G -> A	nhaB	b1186	TCT -> TTT	MISSENSE	S350F	ER2796_1197	
SNP (transition)	1,290,170	1272577 C -> T	rssB	b1235	CAA -> TAA	NONSENSE	Q236stop	ER2796_1251	Removes aa 236-337
SNP (transversion)	1,292,215	1274622 A -> C	–	–	–	INTERGENIC	–	–	
SNP (transversion)	1,300,016	1282423 A -> T	oppA	b1243	AAT -> TAT	MISSENSE	N271Y	ER2796_1262	a
SNP (transversion)	1,304,760	1287167 T -> G	oppF	b1247	TCC -> GCC	MISSENSE	S325A	ER2796_1266	a
SNP (transition)	1,310,701	1293108 C -> T	yciB	b1254	GAG -> AAG	MISSENSE	E72K	ER2796_1274	
SNP (transition)	1,319,610	1302017 C -> T	trpE	b1264	GGT -> GAT	MISSENSE	G454D	ER2796_1284	
SNP (transition)	1,331,687	1314094 T -> C	–	–	–	INTERGENIC	–	–	
SNP (transition)	1,335,418	1317825 A -> G	acnA	b1276	AGC -> GGC	MISSENSE	S522G	ER2796_1297	a
SNP (transition)	1,344,271	1326677 C -> T	gmr	b1285	GCT -> ACT	MISSENSE	A166T	ER2796_1309	
SNP (transition)	1,354,698	1337104 G -> A	sapA	b1294	CCC -> CTC	MISSENSE	P146L	ER2796_1318	
SNP (transition)	1,356,797	1339203 C -> T	puuP	b1296	GTA -> ATA	MISSENSE	V139I	ER2796_1320	
SNP (transition)	1,356,883	1339289 T -> C	puuP	b1296	TAT -> TGT	MISSENSE	Y110C	ER2796_1320	a
SNP (transition)	1,363,791	1346197 C -> T	puuE	b1302	ACC -> ATC	MISSENSE	T73I	ER2796_1328	
SNP (transversion)	1,365,250	1347656 C -> A	pspF	b1303	TTG -> TTT	MISSENSE	L229F	ER2796_1329	b
SNP (transition)	1,369,943	1352349 C -> T	ycjN	b1310	TCA -> TTA	MISSENSE	S4L	ER2796_1336	
SNP (transversion)	1,372,522	1354928 G -> T	ycjP	b1312	GGC -> TGC	MISSENSE	G137C	ER2796_1338	
SNP (transition)	1,403,782	1386188 G -> A	–	–	–	INTERGENIC	–	–	
SNP (transition)	1,427,274	1409679 C -> T	stfR	b1372	CCG -> TCG	MISSENSE	P68S	ER2796_1398	
SNP (transition)	1,431,168	1413573 G -> A	pinR	b1374	TTC -> TTT	SILENT	F177	ER2796_1400	
SNP (transition)	1,443,652	1426057 C -> T	ydbH	b1381	CAG -> TAG	NONSENSE	Q860stop	ER2796_1409	Removes aa 860-879
SNP (transversion)	1,457,197	1439602 G -> T	paaH	b1395	GCG -> GCT	SILENT	A40	ER2796_1424	
SNP (transition)	1,484,325	1466730 C -> T	hrpA	b1413	CCA -> TCA	MISSENSE	P1081S	ER2796_1443	
SNP (transition)	1,529,713	1512118 A -> G	–	–	–	INTERGENIC	–	–	
SNP (transversion)	1,542,750	1525155 G -> C	yddK	b1471	GCC -> GGC	MISSENSE	A330G	–	d
SNP (transversion)	1,549,014	1531419 A -> T	fdnH	b1475	AAA -> ATA	MISSENSE	K177I	ER2796_1510	
SNP (transversion)	1,551,654	1534059 C -> A	adhP	b1478	GGT -> GTT	MISSENSE	G70V	ER2796_1513	
SNP (transition)	1,598,258	1580662 C -> T	–	–	–	INTERGENIC	–	–	

SNP (transversion)	1,599,122	1581526	C -> A	lsrR	b1512	TCG -> TCT	SILENT	S48	ER2796_1549	
SNP (transition)	1,599,127	1581531	C -> T	lsrR	b1512	GTG -> ATG	MISSENSE	V47M	ER2796_1549	
SNP (transition)	1,606,154	1588558	T -> C	yneE	b1520	GAT -> GGT	MISSENSE	D298G	ER2796_1557	
SNP (transition)	1,630,736	1613140	A -> G	–	–	–	INTERGENIC	–	–	e
SNP (transition)	1,630,759	1613163	G -> A	–	–	–	INTERGENIC	–	–	e
SNP (transition)	1,630,794	1613198	T -> C	–	–	–	INTERGENIC	–	–	e
SNP (transition)	1,630,861	1613265	A -> G	–	–	–	INTERGENIC	–	–	e
SNP (transversion)	1,630,937	1613341	A -> T	–	–	–	INTERGENIC	–	–	e
SNP (transversion)	1,630,940	1613344	T -> G	–	–	–	INTERGENIC	–	–	e
SNP (transition)	1,630,973	1613377	C -> T	–	–	–	INTERGENIC	–	–	e
SNP (transversion)	1,631,247	1613651	A -> C	ydfK	b1544	AAA -> ACA	MISSENSE	K51T	ER2796_1584	e
SNP (transition)	1,639,026	1622768	T -> C	–	–	–	INTERGENIC	–	–	
SNP (transversion)	1,641,703	1625445	A -> T	ydfU	b1560	CTG -> CAG	MISSENSE	L209Q	ER2796_1601	a
SNP (transition)	1,646,986	1630728	C -> T	ydfC	b1573	TCC -> TTC	MISSENSE	S47F	ER2796_1610	
SNP (transition)	1,647,995	1631737	C -> T	ydfD	b1576	CCG -> TCG	MISSENSE	P60S	ER2796_1613	
SNP (transition)	1,650,355	1634097	T -> C	intQ	b1579	TTC -> CTC	MISSENSE	F261L	–	d
SNP (transition)	1,669,476	1653218	C -> T	asr	b1597	ACC -> ATC	MISSENSE	T26I	ER2796_1634	
SNP (transition)	1,679,937	1663679	G -> A	ydgC	b1607	CTT -> TTT	MISSENSE	L40F	ER2796_1643	
SNP (transition)	1,700,781	1684523	C -> T	add	b1623	TTC -> TTT	SILENT	F175	ER2796_1660	
SNP (transition)	1,764,633	1748375	G -> A	ydiJ	b1687	CGC -> TGC	MISSENSE	R693C	ER2796_1729	
SNP (transition)	1,789,661	1773403	G -> A	cdgR	b1707	CGC -> CGT	SILENT	R128	ER2796_1749	
SNP (transition)	1,804,097	1787838	A -> G	ydiY	b1722	TTG -> TCG	MISSENSE	L4S	ER2796_1766	
SNP (transition)	1,823,473	1807214	G -> A	spy	b1743	GAC -> GAT	SILENT	D59	ER2796_1787	
SNP (transition)	1,826,853	1810594	G -> A	astD	b1746	AGC -> AGT	SILENT	S302	ER2796_1790	
SNP (transition)	1,872,226	1855967	G -> A	–	–	–	INTERGENIC	–	–	
SNP (transition)	1,890,902	1874643	C -> T	yoaA	b1808	GGC -> AGC	MISSENSE	G120S	ER2796_1855	
SNP (transition)	1,892,863	1876604	T -> C	pabB	b1812	CTC -> CCC	MISSENSE	L12P	ER2796_1860	a
Substitution	1909953-1909954	1893694-1893695	CC -> TT	htpX	b1829	TGG -> TAA	NONSENSE	W216stop	ER2796_1878	Removes aa 216-293
SNP (transition)	1,918,646	1902387	C -> T	rsmF	b1835	CAA -> TAA	NONSENSE	Q134stop	ER2796_1886	Removes aa 134-479
SNP (transition)	1,951,319	1935060	G -> A	cmoA	b1870	AAG -> AAA	SILENT	K198	ER2796_1922	
SNP (transition)	1,985,082	1968047	C -> T	ftnB	b1902	GCC -> GTC	MISSENSE	A45V	ER2796_1953	
SNP (transition)	1,991,357	1974322	C -> T	uvrC	b1913	AAG -> AAA	SILENT	K458	ER2796_1966	
SNP (transition)	2,029,184	2012149	C -> T	dcm	b1961	GAG -> GAA	SILENT	E386	ER2796_2012	
SNP (transition)	2,030,207	2013172	C -> T	dcm	b1961	TGG -> TGA	NONSENSE	W45stop	ER2796_2012	Removes aa 45-472
SNP (transition)	2,031,672	2014637	C -> T	–	–	–	INTERGENIC	–	–	
SNP (transversion)	2,038,457	2021422	C -> A	yedY	b1971	GCC -> GAC	MISSENSE	A319D	ER2796_2024	a
SNP (transition)	2,082,078	2074199	G -> A	sbcB	b2011	CAG -> CAA	SILENT	Q433	ER2796_2075	
SNP (transversion)	2,097,549	2089634	A -> T	ugd	b2028	ATT -> AAT	MISSENSE	I30N	ER2796_2091	
SNP (transition)	2,137,015	2129099	G -> A	yegH	b2063	GAA -> AAA	MISSENSE	E364K	ER2796_2125	
SNP (transition)	2,153,904	2145988	C -> T	mdtB	b2075	CTC -> CTT	SILENT	L206	ER2796_2137	
SNP (transition)	2,171,470	2163554	C -> T	gatC	b2092	CAG -> CAA	SILENT	Q277	ER2796_2154	

SNP (transition)	2,222,191	2214275	A -> G	pbpG	b2134	TTT -> TTC	SILENT	F234	ER2796_2198	
SNP (transversion)	2,235,127	2227211	G -> T	mglC	b2148	CCG -> ACG	MISSENSE	P217T	ER2796_2214	
Substitution	2262482-2262483	2255343-2255344	GA -> AT	setB	b2170	GAG -> ATG	MISSENSE	E200M	ER2796_2240	
SNP (transversion)	2,296,319	2289180	A -> T	napB	b2203	TTT -> TAT	MISSENSE	F141Y	ER2796_2274	
SNP (transversion)	2,317,100	2309961	C -> A	rcsC	b2218	GAT -> TAT	MISSENSE	D267Y	ER2796_2290	
SNP (transversion)	2,390,255	2383115	C -> G	nuoM	b2277	GGT -> GCT	MISSENSE	G270A	ER2796_2353	
SNP (transition)	2,421,913	2414773	C -> T	hisP	b2306	GTA -> ATA	MISSENSE	V207I	ER2796_2383	
SNP (transition)	2,441,469	2434329	G -> A	mnmC	b2324	GCG -> ACG	MISSENSE	A562T	ER2796_2403	
SNP (transition)	2,447,086	2439946	G -> A	smrB	b2331	ATG -> ATA	MISSENSE	M153I	ER2796_2410	
SNP (transition)	2,508,631	2501492	C -> T	yfeO	b2389	CCG -> CTG	MISSENSE	P327L	ER2796_2471	
SNP (transition)	2,585,134	2571205	C -> T	narQ	b2469	GCA -> GTA	MISSENSE	A461V	ER2796_2544	
SNP (transition)	2,628,874	2614945	G -> A	yfgI	b2506	AGC -> AAC	MISSENSE	S176N	ER2796_2584	
SNP (transition)	2,629,418	2615489	G -> A	guaA	b2507	TTC -> TTT	SILENT	F380	ER2796_2585	
SNP (transition)	2,724,405	2710476	C -> T	rrlG	b2589	–	rRNA	–	–	e
SNP (transition)	2,724,408	2710479	T -> C	rrlG	b2590	–	rRNA	–	–	e
SNP (transition)	2,724,411	2710482	A -> G	rrlG	b2591	–	rRNA	–	–	e
SNP (transition)	2,724,413	2710484	G -> A	rrlG	b2592	–	rRNA	–	–	e
SNP (transition)	2,731,014	2717085	G -> A	clpB	b2592	ATC -> ATT	SILENT	I394	ER2796_2675	
SNP (transition)	2,802,155	2788234	C -> T	nrdF	b2676	TCT -> TTT	MISSENSE	S211F	ER2796_2749	
SNP (transition)	2,812,483	2798561	C -> T	luxS	b2687	CTG -> CTA	SILENT	L91	ER2796_2762	
SNP (transversion)	2,847,158	2833236	C -> A	hycC	b2723	GGA -> TGA	NONSENSE	G36stop	ER2796_2802	Removes aa 36-608
SNP (transition)	2,865,477	2851555	G -> A	rpoS	b2741	CAG -> TAG	NONSENSE	Q33stop	ER2796_2821	Removes aa 33-330
SNP (transversion)	2,869,175	2855253	T -> G	truD	b2745	AAC -> ACC	MISSENSE	N51T	ER2796_2826	
SNP (transition)	2,906,979	2893057	G -> A	pyrG	b2780	TCT -> TTT	MISSENSE	S237F	ER2796_2865	
Substitution	2924565-2924566	2910643-2910644	CC -> TT	ygdH	b2795	CCC -> CTT	MISSENSE	P79L	ER2796_2882	
SNP (transversion)	2,924,794	2910872	C -> A	ygdH	b2795	GGC -> GGA	SILENT	G155	ER2796_2882	
SNP (transition)	2,979,946	2966024	C -> T	araE	b2841	GGG -> AGG	MISSENSE	G87R	ER2796_2930	
SNP (transition)	2,980,022	2966100	C -> T	araE	b2841	CAG -> CAA	SILENT	Q61	ER2796_2930	
SNP (transition)	2,983,541	2969619	G -> A	yqeF	b2844	TCC -> TTC	MISSENSE	S25F	ER2796_2933	
SNP (transition)	2,991,235	2977313	C -> T	ygeH	b2852	CTC -> TTC	MISSENSE	L374F	ER2796_2941	
SNP (transition)	3,038,864	3024942	G -> A	cptB	b2897	CAG -> TAG	NONSENSE	Q77stop	ER2796_2983	Removes aa 77-88
SNP (transition)	3,038,865	3024943	G -> A	cptB	b2897	ATC -> ATT	SILENT	I76	ER2796_2983	
SNP (transition)	3,041,382	3027460	G -> A	yqfB	b2900	ATC -> ATT	SILENT	I88	ER2796_2987	
SNP (transition)	3,069,434	3055512	C -> T	–	–	–	INTERGENIC	–	–	
SNP (transversion)	3,087,131	3073209	A -> T	galP	b2943	AAA -> TAA	NONSENSE	K276stop	ER2796_3029	Removes aa 276-464
SNP (transition)	3,130,554	3116632	G -> A	yghR	b2984	CCC -> CCT	SILENT	P227	ER2796_3074	
SNP (transition)	3,159,638	3145716	G -> A	ftsP	b3017	CGC -> TGC	MISSENSE	R352C	ER2796_3108	
SNP (transition)	3,165,588	3151666	C -> T	ygiS	b3020	GTG -> GTA	SILENT	V51	ER2796_3111	
SNP (transition)	3,284,577	3271853	C -> T	agaI	b3141	CCA -> TCA	MISSENSE	P96S	ER2796_3234	
SNP (transversion)	3,327,765	3315040	G -> T	dacB	b3182	GAT -> TAT	MISSENSE	D261Y	ER2796_3276	
SNP (transversion)	3,386,063	3356988	T -> G	aaeB	b3240	ACG -> CCG	MISSENSE	T50P	ER2796_3325	

SNP (transition)	3,390,542	3361467	G -> A	yhdP	b4472	CCG -> TCG	MISSENSE	P1247S	ER2796_3330	
SNP (transversion)	3,422,257	3393182	A -> C	rrlD	b3275	–	rRNA	–	–	a, e
SNP (transversion)	3,422,258	3393183	T -> A	rrlD	b3275	–	rRNA	–	–	a, e
SNP (transition)	3,422,259	3393184	C -> T	rrlD	b3275	–	rRNA	–	–	a, e
SNP (transition)	3,430,127	3401052	C -> T	smg	b3284	CTG -> CTA	SILENT	L120	ER2796_3371	
SNP (transition)	3,441,983	3412908	G -> A	secY	b3300	GCC -> GTC	MISSENSE	A46V	ER2796_3386	
SNP (transition)	3,442,002	3412927	G -> A	secY	b3300	CCG -> TCG	MISSENSE	P40S	ER2796_3386	
SNP (transition)	3,450,044	3420969	G -> A	rplD	b3319	CCG -> TCG	MISSENSE	P89S	ER2796_3405	
SNP (transition)	3,450,240	3421165	G -> A	rplD	b3319	TTC -> TTT	SILENT	F23	ER2796_3405	
SNP (transition)	3,454,055	3424980	T -> C	gspC	b3324	ACT -> ACC	SILENT	T152	ER2796_3410	
SNP (transition)	3,455,400	3426325	C -> T	gspD	b3325	CTC -> CTT	SILENT	L334	ER2796_3411	
SNP (transversion)	3,456,267	3427192	A -> C	gspD	b3325	TCA -> TCC	SILENT	S623	ER2796_3411	
SNP (transversion)	3,472,313	3443238	T -> G	rpsL	b3342	AAA -> CAA	MISSENSE	K88Q	ER2796_3428	
SNP (transversion)	3,472,447	3443372	T -> G	rpsL	b3342	AAA -> ACA	MISSENSE	K43T	ER2796_3428	
SNP (transition)	3,486,778	3457703	G -> A	yhfK	b3358	GCA -> ACA	MISSENSE	A656T	ER2796_3445	
SNP (transition)	3,492,701	3463626	C -> T	nirB	b3365	ACC -> ACT	SILENT	T223	ER2796_3453	
SNP (transition)	3,536,021	3507679	C -> T	yhgF	b3407	TTC -> TTT	SILENT	F205	ER2796_3497	
SNP (transversion)	3,553,069	3524727	G -> C	malT	b3418	GTC -> CTC	MISSENSE	V655L	ER2796_3508	
SNP (transition)	3,604,914	3576572	C -> T	zntA	b3469	TTC -> TTT	SILENT	F147	ER2796_3560	
SNP (transversion)	3,615,402	3587060	T -> A	nikD	b3479	ATA -> AAA	MISSENSE	I122K	ER2796_3570	
SNP (transversion)	3,622,568	3594226	T -> A	yhhI	b3484	GAT -> GAA	MISSENSE	D56E	ER2796_3574	
Substitution	3629379-3629380	3601037-3601038	TT -> AG	yhiJ	b3488	AAT -> CTT	MISSENSE	N412L	ER2796_3579	
SNP (transition)	3,645,451	3617109	C -> T	gor	b3500	GCG -> GTG	MISSENSE	A377V	ER2796_3593	
SNP (transition)	3,658,805	3630463	G -> A	mdtF	b3514	CAG -> CAA	SILENT	Q123	ER2796_3610	
SNP (transition)	3,677,531	3649189	C -> T	kdgK	b3526	ACC -> ACT	SILENT	T30	ER2796_3624	
SNP (transition)	3,684,728	3656386	G -> A	bcsC	b3530	TTC -> TTT	SILENT	F823	ER2796_3629	
SNP (transversion)	3,705,970	3678827	C -> A	–	–	–	INTERGENIC	–	–	
SNP (transition)	3,710,430	3683287	G -> A	yhjY	b3548	GGC -> GGT	SILENT	G176	ER2796_3651	
SNP (transversion)	3,723,199	3696056	T -> G	glyQ	b3560	GAG -> GCG	MISSENSE	E48A	ER2796_3664	a
SNP (transition)	3,726,511	3699368	G -> A	xylB	b3564	GCG -> GTG	MISSENSE	A295V	ER2796_3669	
SNP (transition)	3,727,978	3700835	G -> A	xylA	b3565	CAC -> TAC	MISSENSE	H271Y	ER2796_3670	
SNP (transition)	3,727,979	3700836	G -> A	xylA	b3565	AAC -> AAT	SILENT	N270	ER2796_3670	
SNP (transition)	3,746,801	3720435	G -> A	yiaQ	b3581	GCT -> ACT	MISSENSE	A68T	ER2796_3688	
SNP (transversion)	3,761,159	3734793	A -> C	rhsA	b3593	CCA -> CCC	SILENT	P318	ER2796_3702	
SNP (transition)	3,767,470	3741104	G -> A	yibV	b4615	GAA -> AAA	MISSENSE	E35K	–	d
SNP (transition)	3,782,006	3755638	G -> A	secB	b3609	TCC -> TTC	MISSENSE	S49F	ER2796_3720	
SNP (transition)	3,798,910	3772542	C -> T	rfaY	b3625	GAC -> AAC	MISSENSE	D27N	ER2796_3736	
SNP (transition)	3,863,908	3837541	A -> G	yidE	b3685	GTT -> GCT	MISSENSE	V130A	ER2796_3797	
SNP (transition)	3,897,218	3870851	C -> T	yieK	b3718	GAA -> AAA	MISSENSE	E67K	ER2796_3830	
SNP (transition)	3,901,257	3874890	G -> A	bglB	b3721	GTC -> GTT	SILENT	V156	ER2796_3833	
SNP (transversion)	3,934,223	3907856	G -> C	rbsC	b3750	GTC -> CTC	MISSENSE	V305L	ER2796_3863	

SNP (transition)	3,942,817	3916450 G -> A	rrlC	b3758	–	rRNA	–	–	
SNP (transition)	3,942,917	3916550 G -> A	rrlC	b3759	–	rRNA	–	–	
SNP (transition)	3,957,957	3931590 C -> T	–	–	–	INTERGENIC	–	–	
SNP (transversion)	3,959,685	3933318 G -> T	rep	b3778	GGC -> GTC	MISSENSE	G329V	ER2796_3888	
SNP (transition)	3,963,197	3936830 G -> A	rhlB	b3780	CAC -> TAC	MISSENSE	H153Y	ER2796_3890	
SNP (transition)	3,966,706	3940339 G -> A	rfe	b3784	ATG -> ATA	MISSENSE	M256I	ER2796_3895	
SNP (transition)	3,967,771	3941404 T -> C	wzzE	b3785	TCG -> CCG	MISSENSE	S240P	ER2796_3896	
SNP (transition)	3,991,554	3965187 T -> C	cyaA	b3806	CGT -> CGC	SILENT	R793	ER2796_3919	a
SNP (transversion)	4,033,757	4007390 T -> G	rrsA	b3851	–	rRNA	–	–	a
SNP (transition)	4,164,671	4138302 G -> A	–	–	–	INTERGENIC	–	–	e
SNP (transversion)	4,166,238	4139869 A -> T	–	–	–	INTERGENIC	–	–	e
SNP (transition)	4,166,244	4139875 T -> C	–	–	–	INTERGENIC	–	–	e
SNP (transition)	4,166,495	4140040 G -> A	–	–	–	INTERGENIC	–	–	e
SNP (transversion)	4,166,498	4140043 G -> T	–	–	–	INTERGENIC	–	–	e
SNP (transition)	4,166,499	4140044 A -> G	–	–	–	INTERGENIC	–	–	e
SNP (transition)	4,206,155	4179698 G -> A	–	–	–	INTERGENIC	–	–	
SNP (transition)	4,221,661	4195204 T -> C	–	–	–	INTERGENIC	–	–	
SNP (transition)	4,239,471	4213791 A -> G	xylE	b4031	CGT -> CGC	SILENT	R269	ER2796_4163	
SNP (transversion)	4,276,098	4250418 T -> A	–	–	–	INTERGENIC	–	–	
SNP (transition)	4,294,291	4268611 T -> C	–	–	–	INTERGENIC	–	–	
SNP (transition)	4,294,988	4269197 G -> A	yjcO	b4078	GCC -> GTC	MISSENSE	A54V	ER2796_4217	
SNP (transition)	4,363,457	4337666 G -> A	–	–	–	INTERGENIC	–	–	
SNP (transition)	4,398,277	4372486 C -> T	–	–	–	INTERGENIC	–	–	
SNP (transition)	4,405,349	4379558 G -> A	rnr	b4179	GAT -> AAT	MISSENSE	D225N	ER2796_4325	
SNP (transversion)	4,405,736	4379945 G -> C	rnr	b4179	GTG -> CTG	MISSENSE	V354L	ER2796_4325	
SNP (transition)	4,421,360	4395569 C -> T	ulaE	b4197	TCC -> TCT	SILENT	S164	ER2796_4343	
SNP (transition)	4,447,609	4421818 G -> A	ppa	b4226	CCG -> TCG	MISSENSE	P23S	ER2796_4371	
SNP (transition)	4,464,569	4438778 G -> A	treR	b4241	ACC -> ATC	MISSENSE	T234I	ER2796_4386	
SNP (transition)	4,471,923	4446132 C -> T	yjgI	b4249	GGA -> AGA	MISSENSE	G52R	ER2796_4392	
SNP (transition)	4,473,516	4447725 T -> C	yjgL	b4253	TTT -> TTC	SILENT	F19	ER2796_4395	
SNP (transition)	4,491,121	4465330 C -> T	idnO	b4266	GGC -> GAC	MISSENSE	G85D	ER2796_4409	b
SNP (transversion)	4,508,261	4482470 T -> G	–	–	–	INTERGENIC	–	–	
SNP (transition)	4,610,121	4529109 G -> A	–	–	–	INTERGENIC	–	–	
SNP (transition)	4,610,810	4529798 G -> A	yjjU	b4377	CGG -> CAG	MISSENSE	R126Q	ER2796_4486	
SNP (transition)	4,613,682	4532670 A -> G	yjjI	b4380	CGT -> CGC	SILENT	R469	ER2796_4489	
SNP (transition)	4,616,579	4535567 C -> T	deoA	b4382	CCG -> TCG	MISSENSE	P110S	ER2796_4493	
SNP (transition)	4,620,335	4539323 C -> T	yjjJ	b4385	CTC -> TTC	MISSENSE	L182F	ER2796_4496	
SNP (transition)	4,634,065	4553053 G -> A	creB	b4398	GAG -> GAA	SILENT	E12	ER2796_4510	
SNP (transversion)	4,634,948	4553936 G -> C	creC	b4399	CGC -> CCC	MISSENSE	R77P	ER2796_4511	
SNP (transition)	4,635,315	4554303 C -> T	creC	b4399	GGC -> GGT	SILENT	G199	ER2796_4511	
SNP (transition)	4,636,263	4555251 G -> A	creD	b4400	TTG -> TTA	SILENT	L21	ER2796_4512	

SNP (transition)	4,637,554	4556542 G -> A	–	–	–	INTERGENIC	–	–

Footnotes

a	Shared with DH10b
b	Not in ER3413
c	Same mutation affects both overlapping ORFs
d	MG1655 pseudogene
e	These consecutive changes (orange shading) from a single gene conversion event
