# Supplementary material for: Complete Genome Sequence of ER2796, a DNA Methyltransferase-Deficient Strain of Escherichia coli K-12
Source: PLoS One. 2015 May 26;10(5):e0127446. doi: 10.1371/journal.pone.0127446 (PMC4444293; doi:10.1371/journal.pone.0127446)
Supplement: S2 Table — (A) Disrupted ORFs. (B) ORFs with missense mutations. (PDF) [file pone.0127446.s003.pdf]

Table S2A. List of ORFs with nonsynonymous changes from the reference strain MG1655 to ER2796 (disrupted ORFs).

MG1655 Gene	MG1655 Locus	ER2796 Gene	ER2796 Locus	ER2796 Start..Stop	Disruption	Intervening ORFs	MG1655 Functional Description
fhuA	b0150	fhuA	ER2796_149/151	167484..171063	IS2 insertion	ER2796_148, ER2796_150	ferrichrome outer membrane transporter
rclA	b0304	rclA	ER2796_311/–	321899..319236	IS10 insertion	ER2796_310	putative pyridine nucleotide-disulfide oxidoreductase
lacZ	b0344	lacZ	ER2796_352	365759..364971	deletion	–	beta-D-galactosidase
ybcV	b0558	ybcV	–	575649..575239	deletion	–	DLP12 prophage; putative protein
nohD	b0560	nohD	ER2796_–/567	576889..578211	IS1 insertion	ER2796_565, ER2796_566	DLP12 prophage; DNA packaging protein
yliE	b0833	yliE	ER2796_–/851	869811..872158	frameshift	–	putative membrane-anchored cyclic-di-GMP phosphodiesterase
rssB	b1235	rssB	ER2796_1251/1252	1271872..1272885	nonsense	–	response regulator binding RpoS to initiate proteolysis by ClpXP; required for the PcnB-degradosome interaction during stationary phase
yciT	b1284	yciT	ER2796_1307/1306	1324776..1324028	frameshift	–	global regulator of transcription; DeoR family
ydbH	b1381	ydbH	ER2796_1409/–	1423480..1426119	nonsense	–	hypothetical protein
btuD	b1709	btuD	ER2796_1752/1751	1775323..1774575	frameshift	–	vitamin B12 transporter subunit : ATP-binding component of ABC superfamily
htpX	b1829	htpX	ER2796_1879/1878	1894341..1893460	nonsense	–	putative endopeptidase
rsmF	b1835	rsmF	ER2796_1885/1886	1901988..1903427	nonsense	–	16S rRNA m(5)C1407 methyltransferase, SAM-dependent
dcm	b1961	dcm	ER2796_2013/2012	2013306..2011888	nonsense	–	DNA cytosine methyltransferase
yedZ	b1972	yedZ	ER2796_2025/2035	2021472..2031263	Tn10 insertion	ER2796_2026 to _2034	inner membrane heme subunit for periplasmic YedYZ reductase
hisG	b2019	hisG	ER2796_2082	2080337..2081200	deletion	–	ATP phosphoribosyltransferase
rfbD	b2040	rfbD	ER2796_2102/–	2102084..2101186	frameshift	–	dTDP-4-dehydrorhamnose reductase subunit, NAD(P)-binding, of dTDP-L-rhamnose synthase
mglA	b2149	mglA	ER2796_2216/–	2230172..2227875	IS1 insertion	ER2796_2215	fused methyl-galactoside transporter subunits of ABC superfamily: ATP-binding components
rhmD	b2247	rhmD	ER2796_2322/2321	2352296..2351092	frameshift	–	L-rhamnonate dehydratase
eutA	b2451	eutA	ER2796_2526	2550977..2549574	deletion	–	reactivating factor for ethanolamine ammonia lyase
luxS	b2687	luxS	ER2796_2763/2762	2798833..2798319	frameshift	–	S-ribosylhomocysteine lyase
hycC	b2723	hycC	ER2796_2803/2802	2833341..2831515	nonsense	–	hydrogenase 3, membrane subunit
rpoS	b2741	rpoS	ER2796_–/2821	2851651..2850659	nonsense	–	RNA polymerase, sigma S (sigma 38) factor
cptB	b2897	cptB	ER2796_2983/–	3025170..3024904	nonsense	–	antitoxin of CptAB toxin-antitoxin pair
galP	b2943	galP	ER2796_3029/3030	3072384..3073778	nonsense	–	D-galactose transporter
ttdB	b3062	ttdB	ER2796_3151/3152	3191471..3192075	frameshift	–	L-tartrate dehydratase, beta subunit
tdcD	b3115	tdcD	ER2796_3208/3206	3248958..3246551	IS5 insertion	ER2796_3207	propionate kinase/acetate kinase C, anaerobic
argG	b3172	argG	ER2796_3265/3266	3303935..3305277	frameshift	–	argininosuccinate synthetase
dam	b3387	dam	ER2796_–/3474	3485593..3484024	deletion, KanR insertion	ER2796_3475	DNA adenine methyltransferase
xylF	b3566	xylF	ER2796_3671/3673	3702011..3703780	IS1 insertion	ER2796_3672	D-xylose transporter subunit
mtlA	b3599	mtlA	ER2796_3708/3709	3743938..3745849	frameshift	–	fused mannitol-specific PTS enzymes: IIA components/IIB components/IIC components
rph	b3643	rph	ER2796_3754/–	3788205..3787518	frameshift	–	defective ribonuclease PH
metB	b3939	metB	ER2796_–/4061	4100328..4101486	frameshift	–	cystathionine gamma-synthase, PLP-dependent
opgB	b4359	opgB	ER2796_4466	4516451..4514439	deletion, IS10 insertion	–	phosphoglycerol transferases I and II

Table S2B. List of ORFs with nonsynonymous changes from the reference strain MG1655 to ER2796 (ORFs with missense mutations).

MG1655 Gene	MG1655 Locus	ER2796 Gene	ER2796 Locus	Codon Change(s)	Consequence(s)	MG1655 Functional Description
rpsT	b0023	rpsT	ER2796_18	GCC -> GTC	A11V	30S ribosomal subunit protein S20
ileS	b0026	ileS	ER2796_20	GAT -> AAT	D510N	isoleucyl-tRNA synthetase
yadV	b0140	yadV	ER2796_137	GTA -> GCA	V45A	putative periplasmic pilin chaperone
yafJ	b0223	yafJ	ER2796_228	GGC -> AGC	G45S	putative amidotransfease
ecpD	b0290	ecpD	ER2796_297	TCC -> TTT	S202F	putative receptor
cynX	b0341	cynX	ER2796_349	GGC -> GAC	G348D	putative cyanate transporter
clpX	b0438	clpX	ER2796_448	GAT -> AAT	D255N	ATPase and specificity subunit of ClpX-ClpP ATP-dependent serine protease
lon	b0439	lon	ER2796_449	TCT -> TTT	S182F	DNA-binding ATP-dependent protease La
ylbA	b0515	ylbA	ER2796_524	TAA -> CAA	stop262Q	hypothetical protein
entA	b0596	entA	ER2796_607	AAT -> GAT	N7D	2,3-dihydro-2,3-dihydroxybenzoate dehydrogenase
ybiT	b0820	ybiT	ER2796_838	CCT -> TCT; ACG -> AAG	P158S; T360K	fused predicted transporter subunits of ABC superfamily: ATP-binding components
rimK	b0852	rimK	ER2796_870	GAA -> AAA	E178K	ribosomal protein S6 modification protein
rlmC	b0859	rlmC	ER2796_878	CGT -> CAT	R143H	23S rRNA m(5)U747 methyltransferase, SAM-dependent
rpsA	b0911	rpsA	ER2796_932	CGT -> TGT	R171C	30S ribosomal subunit protein S1
smtA	b0921	smtA	ER2796_942	TCC -> TTC	S14F	putative S-adenosyl-L-methionine-dependent methyltransferase
uup	b0949	uup	ER2796_972	GAA -> AAA	E553K	fused predicted transporter subunits of ABC superfamily: ATP-binding components
yceF	b1087	yceF	ER2796_1115	GTT -> TTT	V173F	Maf-like protein
ptsG	b1101	ptsG	ER2796_1130	GTC -> TTC	V12F	fused glucose-specific PTS enzymes: IIB component/IIC component
ycfS	b1113	ycfS	ER2796_1142	CTG -> CCG	L180P	L,D-transpeptidase linking Lpp to murein
icd	b1136	icd	ER2796_1166	GAT -> GAA	D398E	e14 prophage; isocitrate dehydrogenase, specific for NADP+
nhaB	b1186	nhaB	ER2796_1197	TCT -> TTT	S350F	sodium:proton antiporter
oppA	b1243	oppA	ER2796_1262	AAT -> TAT	N271Y	oligopeptide transporter subunit
oppF	b1247	oppF	ER2796_1266	TCC -> GCC	S325A	oligopeptide transporter subunit
yciB	b1254	yciB	ER2796_1274	GAG -> AAG	E72K	putative inner membrane protein
trpE	b1264	trpE	ER2796_1284	GGT -> GAT	G454D	component I of anthranilate synthase
acnA	b1276	acnA	ER2796_1297	AGC -> GGC	S522G	aconitate hydratase 1
gmr	b1285	gmr	ER2796_1309	GCT -> ACT	A166T	cyclic-di-GMP phosphodiesterase; csgD regulator; modulator of Rnase II stability
sapA	b1294	sapA	ER2796_1318	CCC -> CTC	P146L	antimicrobial peptide transport ABC transporter periplasmic binding protein
puuP	b1296	puuP	ER2796_1320	TAT -> TGT; GTA -> ATA	Y110C; V139I	putrescine importer
puuE	b1302	puuE	ER2796_1328	ACC -> ATC	T73I	GABA aminotransferase, PLP-dependent
pspF	b1303	pspF	ER2796_1329	TTG -> TTT	L229F	DNA-binding transcriptional activator
ycjN	b1310	ycjN	ER2796_1336	TCA -> TTA	S4L	putative sugar transporter subunit: periplasmic-binding component of ABC superfamily
ycjP	b1312	ycjP	ER2796_1338	GGC -> TGC	G137C	putative sugar transporter subunit: membrane component of ABC superfamily
stfR	b1372	stfR	ER2796_1398	CCG -> TCG	P68S	Rac prophage; predicted tail fiber protein
hrpA	b1413	hrpA	ER2796_1443	CCA -> TCA	P1081S	putative ATP-dependent helicase
fdnH	b1475	fdnH	ER2796_1510	AAA -> ATA	K177I	formate dehydrogenase-N, Fe-S (beta) subunit, nitrate-inducible
adhP	b1478	adhP	ER2796_1513	GGT -> GTT	G70V	ethanol-active dehydrogenase/acetaldehyde-active reductase
lsrR	b1512	lsrR	ER2796_1549	GTG -> ATG	V47M	lsr operon transcriptional repressor
yneE	b1520	yneE	ER2796_1557	GAT -> GGT	D298G	putative inner membrane protein, bestrophin family
ydfK	b1544	ydfK	ER2796_1584	AAA -> ACA	K51T	cold shock protein, function unknown, Qin prophage
ydfU	b1560	ydfU	ER2796_1601	CTG -> CAG	L209Q	Qin prophage; putative protein
ydfC	b1573	ydfC	ER2796_1610	TCC -> TTC	S47F	conserved protein, Qin prophage
ydfD	b1576	ydfD	ER2796_1613	CCG -> TCG	P60S	Qin prophage; putative protein
asr	b1597	asr	ER2796_1634	ACC -> ATC	T26I	acid shock-inducible periplasmic protein
ydgC	b1607	ydgC	ER2796_1643	CTT -> TTT	L40F	inner membrane protein, GlpM family
ydiJ	b1687	ydiJ	ER2796_1729	CGC -> TGC	R693C	putative FAD-linked oxidoreductase
ydiY	b1722	ydiY	ER2796_1766	TTG -> TCG	L4S	putative outer membrane protein, acid-inducible
yoaA	b1808	yoaA	ER2796_1855	GGC -> AGC	G120S	conserved protein with nucleoside triphosphate hydrolase domain
pabB	b1812	pabB	ER2796_1860	CTC -> CCC	L12P	aminodeoxychorismate synthase, subunit I
ftnB	b1902	ftnB	ER2796_1953	GCC -> GTC	A45V	ferritin B, probable ferrous iron reservoir
yedY	b1971	yedY	ER2796_2024	GCC -> GAC	A319D	membrane-anchored, periplasmic TMAO, DMSO reductase

ugd	b2028	ugd	ER2796_2091	ATT -> AAT	I30N	UDP-glucose 6-dehydrogenase
yegH	b2063	yegH	ER2796_2125	GAA -> AAA	E364K	inner membrane protein
mglC	b2148	mglC	ER2796_2214	CCG -> ACG	P217T	methyl-galactoside transporter subunit
setB	b2170	setB	ER2796_2240	GAG -> ATG	E200M	lactose/glucose efflux system
napB	b2203	napB	ER2796_2274	TTT -> TAT	F141Y	nitrate reductase, small, cytochrome C550 subunit, periplasmic
rcsC	b2218	rcsC	ER2796_2290	GAT -> TAT	D267Y	hybrid sensory kinase in two-component regulatory system with RcsB and YojN
nuoM	b2277	nuoM	ER2796_2353	GGT -> GCT	G270A	NADH:ubiquinone oxidoreductase, membrane subunit M
hisP	b2306	hisP	ER2796_2383	GTA -> ATA	V207I	histidine/lysine/arginine/ornithine transporter subunit
mnmC	b2324	mnmC	ER2796_2403	GCG -> ACG	A562T	fused 5-methylaminomethyl-2-thiouridine-forming enzyme methyltransferase and FAD-dependent demodification enzyme
smrB	b2331	smrB	ER2796_2410	ATG -> ATA	M153I	putative DNA endonuclease
yfeO	b2389	yfeO	ER2796_2471	CCG -> CTG	P327L	putative ion channel protein
narQ	b2469	narQ	ER2796_2544	GCA -> GTA	A461V	sensory histidine kinase in two-component regulatory system with NarP (NarL)
yfgI	b2506	yfgI	ER2796_2584	AGC -> AAC	S176N	hypothetical protein
nrdF	b2676	nrdF	ER2796_2749	TCT -> TTT	S211F	ribonucleoside-diphosphate reductase 2, beta subunit, ferritin-like protein
truD	b2745	truD	ER2796_2826	AAC -> ACC	N51T	tRNA(Glu) pseudouridine(13) synthase
pyrG	b2780	pyrG	ER2796_2865	TCT -> TTT	S237F	CTP synthetase
ygdH	b2795	ygdH	ER2796_2882	CCC -> CTT	P79L	conserved protein, UPF0717 family
araE	b2841	araE	ER2796_2930	GGG -> AGG	G87R	arabinose transporter
yqeF	b2844	yqeF	ER2796_2933	TCC -> TTC	S25F	putative acyltransferase
ygeH	b2852	ygeH	ER2796_2941	CTC -> TTC	L374F	predictedtranscriptional regulator
ftsP	b3017	ftsP	ER2796_3108	CGC -> TGC	R352C	septal ring component that protects the divisome from stress; multicopy suppressor of ftsI(Ts)
agaI	b3141	agaI	ER2796_3234	CCA -> TCA	P96S	galactosamine-6-phosphate isomerase
dacB	b3182	dacB	ER2796_3276	GAT -> TAT	D261Y	D-alanyl-D-alanine carboxypeptidase
aaeB	b3240	aaeB	ER2796_3325	ACG -> CCG	T50P	p-hydroxybenzoic acid efflux system component
secY	b3300	secY	ER2796_3386	CCG -> TCG; GCC -> GTC	P40S; A46V	preprotein translocase membrane subunit
rplD	b3319	rplD	ER2796_3405	CCG -> TCG	P89S	50S ribosomal subunit protein L4
rpsL	b3342	rpsL	ER2796_3428	AAA -> ACA; AAA -> CAA	K43T; K88Q	30S ribosomal subunit protein S12
yhfK	b3358	yhfK	ER2796_3445	GCA -> ACA	A656T	conserved inner membrane protein
malT	b3418	malT	ER2796_3508	GTC -> CTC	V655L	DNA-binding transcriptional activator for the mal regulon and maltotriose-ATP-binding protein
nikD	b3479	nikD	ER2796_3570	ATA -> AAA	I122K	nickel transporter subunit
yhhI	b3484	yhhI	ER2796_3574	GAT -> GAA	D56E	putative transposase
yhiJ	b3488	yhiJ	ER2796_3579	AAT -> CTT	N412L	hypothetical protein
gor	b3500	gor	ER2796_3593	GCG -> GTG	A377V	glutathione oxidoreductase
glyQ	b3560	glyQ	ER2796_3664	GAG -> GCG	E48A	glycine tRNA synthetase, alpha subunit
xylB	b3564	xylB	ER2796_3669	GCG -> GTG	A295V	xylulokinase
xylA	b3565	xylA	ER2796_3670	CAC -> TAC	H271Y	D-xylose isomerase
yiaQ	b3581	yiaQ	ER2796_3688	GCT -> ACT	A68T	3-keto-L-gulonate 6-phosphate decarboxylase
secB	b3609	secB	ER2796_3720	TCC -> TTC	S49F	protein export chaperone
waaY	b3625	waaY	ER2796_3736	GAC -> AAC	D27N	lipopolysaccharide core biosynthesis protein
yidE	b3685	yidE	ER2796_3797	GTT -> GCT	V130A	putative transporter
yieK	b3718	yieK	ER2796_3830	GAA -> AAA	E67K	putative 6-phosphogluconolactonase
rbsC	b3750	rbsC	ER2796_3863	GTC -> CTC	V305L	D-ribose transporter subunit
rep	b3778	rep	ER2796_3888	GGC -> GTC	G329V	DNA helicase and single-stranded DNA-dependent ATPase
rhlB	b3780	rhlB	ER2796_3890	CAC -> TAC	H153Y	ATP-dependent RNA helicase
wecA	b3784	wecA	ER2796_3895	ATG -> ATA	M256I	UDP-GlcNAc:undecaprenylphosphate GlcNAc-1-phosphate transferase
wzzE	b3785	wzzE	ER2796_3896	TCG -> CCG	S240P	Entobacterial Common Antigen (ECA) polysaccharide chain length modulation protein
yjcO	b4078	yjcO	ER2796_4217	GCC -> GTC	A54V	hypothetical protein
rnr	b4179	rnr	ER2796_4325	GAT -> AAT; GTG -> CTG	D225N; V354L	exoribonuclease R, RNase R
ppa	b4226	ppa	ER2796_4371	CCG -> TCG	P23S	inorganic pyrophosphatase
treR	b4241	treR	ER2796_4386	ACC -> ATC	T234I	DNA-binding transcriptional repressor
bdcA	b4249	bdcA	ER2796_4392	GGA -> AGA	G52R	c-di-GMP-binding biofilm dispersal mediator protein
idnO	b4266	idnO	ER2796_4409	GGC -> GAC	G85D	5-keto-D-gluconate-5-reductase
yjjU	b4377	yjjU	ER2796_4486	CGG -> CAG	R126Q	putative phospholipase, patatin-like family
deoA	b4382	deoA	ER2796_4493	CCG -> TCG	P110S	thymidine phosphorylase
yjjJ	b4385	yjjJ	ER2796_4496	CTC -> TTC	L182F	putative protein kinase

creC	b4399	creC	ER2796_4511	CGC -> CCC	R77P	sensory histidine kinase in two-component regulatory system with CreB or PhoB, regulator of the CreBC regulon
yhdP	b4472	yhdP	ER2796_3330	CCG -> TCG	P1247S	conserved membrane protein, predicted transporter
insA	b4516	insA	ER2796_4627	TCA -> TTA	S87L	IS1 repressor TnpA
