# Supplementary material for: Complete Genome Sequence of ER2796, a DNA Methyltransferase-Deficient Strain of Escherichia coli K-12
Source: PLoS One. 2015 May 26;10(5):e0127446. doi: 10.1371/journal.pone.0127446 (PMC4444293; doi:10.1371/journal.pone.0127446)
Supplement: S3 Table — (PDF) [file pone.0127446.s004.pdf]

Table 3. List of additional sequence changes from the reference strain MG1655 to ER3413 over and above those in ER2796. [a]

Type	MG1655 Location	ER2796 Location	Change	Gene	Locus	Codon Change	Type	AA Change	ER2796 ORF	Footnotes
SNP (transition)	98387	98387	C -> T	murD	b0088	GCG -> GTG	MISSENSE	A434V	ER2796_0085	
SNP (transition)	271112	272448	A -> G	–	–	–	INTERGENIC	–	–	
SNP (transition)	1110582	1108371	C -> T	opgH	b1049	GCG -> GTG	MISSENSE	A166V	ER2796_1076	
SNP (transition)	–	1617874	C -> T	–	–	GTT -> ATT	MISSENSE	V266I	ER2796_1591	b
SNP (transition)	–	1618352	T -> C	–	–	CGA -> CGG	SILENT	R106	ER2796_1591	b
SNP (transition)	–	1618367	A -> G	–	–	ATT -> ATC	SILENT	I101	ER2796_1591	b
SNP (transversion)	–	1618574	T -> G	–	–	GCA -> GCC	SILENT	A32	ER2796_1591	b
SNP (transition)	–	1618670	C -> T	–	–	–	INTERGENIC	–	–	b
SNP (transversion)	–	1618694	C -> G	–	–	–	INTERGENIC	–	–	b
SNP (transition)	–	1618730	C -> T	–	–	–	INTERGENIC	–	–	b
SNP (transversion)	–	1618750	T -> G	–	–	–	INTERGENIC	–	–	b
Substitution	–	1618752	CC -> TA	–	–	–	INTERGENIC	–	–	b
SNP (transition)	1913499	1897240	A -> G	proQ	b1831	CGT -> CGC	SILENT	R20	ER2796_1881	
SNP (transition)	3325707	3312982	A -> G	–	–	–	INTERGENIC	–	–	
insertion	bet 3410356-3410357	bet 3381281-3381282	ins CCGG	yhdJ	b3262	–	FRAMESHIFT	–	ER2796_3347	c
insertion	–	3485404	ins C	–	–	–	INTERGENIC	–	–	d
SNP (transition)	3766440	3740074	T -> C	yibG	b3596	TTG -> CTG	SILENT	L81	ER2796_4597	
SNP (transition)	4245102	4219422	G -> A	malK	b4035	GGC -> GAC	MISSENSE	G99D	ER2796_4167	

Footnotes

a	Six changes to MG1655 in ER2796 are not present in ER3413; these are noted in Table S1.
b	Probable gene conversion event in IS10 insertion
c	Engineered disruption
d	Within the insertion at dam, but outside the aph ORF
